# Optimization of Azidophenylselenylation of Glycals for the Efficient Synthesis of Phenyl 2-Azido-2-Deoxy-1-Selenoglycosides: Solvent Control

**DOI:** 10.3390/molecules31010054

**Published:** 2025-12-23

**Authors:** Bozhena S. Komarova, Olesia V. Belova, Timur M. Volkov, Dmitry V. Yashunsky, Nikolay E. Nifantiev

**Affiliations:** Laboratory of Glycoconjugate Chemistry, N.D. Zelinsky Institute of Organic Chemistry, Russian Academy of Sciences, Leninsky Prospect 47, Moscow 119991, Russiaolesya01belova@mail.ru (O.V.B.);

**Keywords:** azidophenylselenylation, glycal, phenylseleno 2-azido-2-deoxy monosaccharide, solvent effect, mass transfer, heterogeneous reaction

## Abstract

Azidophenylselenylation (APS) of glycals is a straightforward transformation for preparing phenylseleno 2-azido-2-deoxy derivatives, which are useful blocks in the synthesis of 2-amino-2-deoxy-glycoside-containing oligosaccharides. However, the previously developed APS methods employing the CH_2_Cl_2_ as solvent, Ph_2_Se_2_-PhI(OAc)_2_ (commonly known as BAIB), and a source of N_3_^−^ are still not universal and show limited efficiency for glycals with *gluco*- and *galacto*-configurations. To address this limitation, we revisited both heterogeneous (using NaN_3_) and homogeneous (using TMSN_3_) APS approaches and optimized the reaction conditions. We found that glycal substrates with *galacto*- and *gluco*-configurations require distinct conditions. *Galacto*-substrates react relatively rapidly, and their conversion depends mainly on efficient azide-ion transfer into the organic phase, which is promoted by nitrile solvents (CH_3_CN, EtCN). In contrast, for the slower *gluco*-configured substrates, complete conversion requires a non-polar solvent still capable of azide-ion transfer, such as benzene. These observations were applied to the optimized synthesis of phenylseleno 2-azido-2-deoxy derivatives of d-galactose, d-glucose, l-fucose, l-quinovose, and l-rhamnose.

## 1. Introduction

1-Azido-2-deoxy derivatives of monosaccharides are widely used as synthetic precursors of 2-amino-2-deoxy-monosaccharides, particularly in the construction of diverse biologically important oligosaccharides containing such units [1,2,3,4,5,6,7]. To introduce 2-azido-2-deoxy-glycosyl residues into oligosaccharide chains, several types of glycosyl donors have been employed, starting from 2-azido-2-deoxy-galactosyl bromides whose pioneering synthesis was proposed by Lemieux and Ratcliffe [8]. Other types of glycosyl donors include glycosyl chlorides [9,10,11], imidates [12,13,14], thioglycosides [15,16,17], hemiacetals [18], and selenoglycosides [19,20,21,22].

**Scheme 1:**
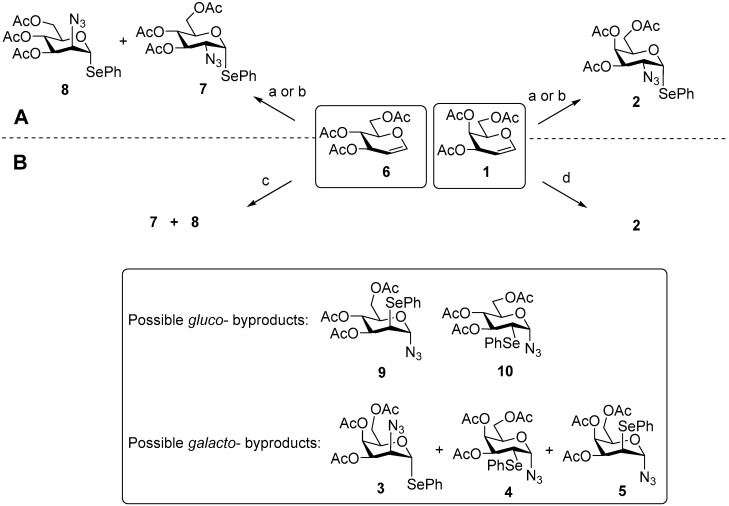
Previously described (**A**) and present (**B**) APS-protocols for galactal **1** and glucal **6** transformation leading to phenylseleno 2-azido-2-deoxyglycosides **2** and **7**, **8** and associated byproducts **3**–**5** and **9**, **10**. Reagents and conditions: (a) [19] TMSN_3_, Ph_2_Se_2_, BAIB, CH_2_Cl_2_; (b) [38] NaN_3_, Ph_2_Se_2_, BAIB, CH_2_Cl_2_; (c) NaN_3_, Ph_2_Se_2_, BAIB, C_6_H_6_; d) NaN_3_, Ph_2_Se_2_, BAIB, EtCN.

2-Azido-2-deoxy-derivatives of phenyl 1-selenoglycosides are regarded as promising glycosylation agents [21,22,23,24,25,26,27,28,29,30,31], as the PhSe leaving group in glycosylation reactions remains stable under typical protecting-group manipulations used in oligosaccharide synthesis. They can be obtained from glycals via anti-Markovnikov addition to the double bond employing Ph_2_Se_2_, NaN_3_, and bis(acetoxy)iodobenzene (BAIB) in CH_2_Cl_2_ [32], with subsequent adaptations of this reaction to glycals by Czernecki [33,34] and Santoyo-González [35], which improved the preparation of 2-azidoselenogalactoside **2** from tri-*O*-acetyl-d-galactal **1** and 2-azidoselenoglucoside **7** from tri-*O*-acetyl-d-glucal **6** (Scheme 1).

The synthesis of phenyl 2-azido-2-deoxy-1-selenoglycosides was significantly improved when homogeneous conditions for the azidophenylselenylation (APS) of glycals using the TMSN_3_-Ph_2_Se_2_-BAIB system were established by us [19,36]. However, both variants of the APS reaction, homogeneous and heterogeneous, remain challenging to scale up and show, in some cases, poor reproducibility. For example, galactal **1** (0.1 mmol) treated with APS/NaN_3_ affords product **2** in 88–90% yield, whereas scaling up to >4 mmol reduces the yield to 53% [19]. In contrast to industrial settings, where hydrodynamics and mass-transfer parameters can be rigorously controlled, laboratory-scale equipment does not allow faithful geometric or mixing similarity upon scale-up. The use of flow conditions [37] for the APS/TMSN_3_ variant provides reproducibility of the process but requires specialized and non-standard equipment, which seems excessive for the synthesis of such a building block.

Another limitation concerns the stereoselectivity of N_3_ addition at C-2. In the case of substrates bearing an equatorial substituent at C-4, such as glucal or rhamnal, the reaction affords a mixture of products with equatorial (*gluco*-configuration) and axial (*manno*-configuration) N_3_ groups in approximately equal amounts [38]. In contrast, for substrates possessing an axial substituent at C-4, such as galactal and fucal, the ratio is markedly shifted toward the C-2-equatorial product with *galacto*-configuration.

Despite extensive studies, a clear understanding of how mixing regime and solvent effects govern the outcome of APS reactions remains limited, which complicates the rational optimization of this transformation. The heterogeneous variant remains highly attractive due to the ready availability of all required reagents and its overall simplicity. This inherent limitation of APS in standard batch reactors was a key motivation for our study, prompting us to identify other experimentally accessible and simple factors that could enable reliable preparative-scale synthesis. Specifically, we systematically examined the influence of solvents with different abilities to dissolve NaN_3_, mass-transfer efficiency, and phase-transfer additives on the outcome of APS of glycals.

The optimized conditions were subsequently applied to the scalable synthesis of the most practically useful phenyl 2-azido-2-deoxy-1-selenoglycosides, including galacto-, fuco- [39], quinovo-, and rhamno-derived building blocks. Particular attention was given to achieving reproducible gram-scale reactions and obtaining the gluco-configured product selectively in the case of glucal substrates.

## 2. Results and Discussion

### 2.1. Reaction Background

**Figure 1:**
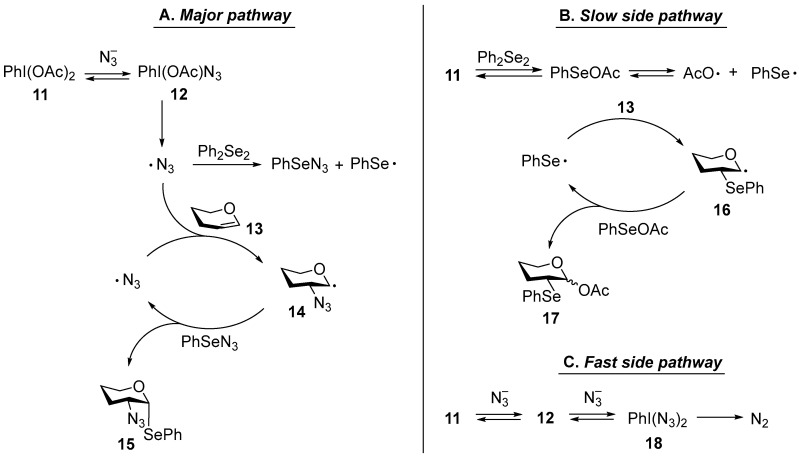
Critical steps of APS mechanism: (**A**) pathway leading to desired 2-azido-2-deoxy-1-seleno-glycosides; (**B**) side pathway resulting in acetyl 2-deoxy-2-seleno-glycosides; (**C**) side pathway connected with N_2_ evolution and azide depletion.

The APS reaction is initiated by ligand exchange of an acetate group at the iodine atom in BAIB (**11**) with N_3_^−^ (Figure 1A) [36], producing a mixed iodane **12**. Subsequent homolytic cleavage of the I-N_3_ bond in **12** generates an azidyl radical (N_3_·), which reacts with Ph_2_Se_2_, forming phenylselenyl azide (PhSeN_3_). PhSeN_3_ is the principal reactive species responsible for the formation of anti-Markovnikov product **15** from the APS-processing of substrate **13** through radical **14**. The overall outcome of the reaction is strongly affected by mass-transfer limitations [40], often leading to diverse and sometimes counterintuitive outcomes. Inadequate agitation of solid NaN_3_ promotes a competing pathway (Figure 1B) [41], giving rise to 2-selenylated radical **16** and subsequently producing acetate **17** [38,42].

Conversely, overly vigorous stirring can disrupt micromixing and foster unproductive radical-radical encounters (e.g., between N_3_ or bis-azide iodane **18**), resulting in N_2_ evolution and azide depletion and thus reducing the effective azide concentration and preventing complete glycal conversion (by analogy with other mixing-sensitive fast radical reactions [43]) (Figure 1C). Polar solvents, while improving NaN_3_ solubility, shift the APS reaction toward Markovnikov selectivity and the formation of 2-phenylseleno derivatives. Consequently, the efficiency of APS depends mainly on three interrelated parameters—solvent polarity, mixing intensity, and the presence of silyl additives (TMSCl or TMSOTf), which promote in situ generation of TMSN_3_ and facilitate phase transfer. To systematically evaluate the impact of these parameters, we first established a set of unified experimental conditions ensuring reproducible mixing and temperature control.

### 2.2. Evaluation of Reaction Parameters on a Small Scale

To systematically evaluate the influence of reaction parameters, we first examined the APS of tri*-O-*acetyl-d-galactal **1** [44] as a model reaction under rigorously controlled conditions (Scheme 1). The preparative synthesis of galactal **1** is described in the Supplementary Information. To ensure reproducible mixing, all APS experiments on a small scale (0.14 and 0.42 mmol per 2 mL; see Tables S1, S3 and S4 in Supplementary Information) were conducted under identical conditions: 7 mL glass tubes (inner diameter 12 mm, PTFE-lined screw caps) equipped with identical 9.5 mm × 4.7 mm PTFE-coated stir bars, stirred at 900 rpm, and thermostatted at 15 ± 0.5 °C—the temperature identified as optimal in preliminary trials. Although no strong sensitivity of APS to moisture or atmosphere was detected, reactions were carried out under argon to avoid oxygen capture and potential impact on byproduct pathways under vigorous mixing.

A wide range of heterogeneous APS conditions is reported, with varying NaN_3_, BAIB, and Ph_2_Se_2_ ratios for different substrates. Particular attention should be given to Ph_2_Se_2_: while its excess ensures a sufficient concentration of the active azide (PhSeN_3_) until full conversion of **13**, the optimal amount differs between sub-0.25 mmol (0.6 equiv.) and >2 mmol (1.2 equiv.) scales [38]. Large excesses, however, reduce the yield and hinder product crystallization [37]. We therefore first examined the influence of substrate **1** concentration (0.07–0.21 M) and the **1** to Ph_2_Se_2_ ratio (0.6–1.2 equiv.) in various solvents to establish a baseline for further optimization (Table S1 in Supplementary Information). These experiments established that, for galactal APS, a concentration of 0.21 M and 0.6 equiv. of Ph_2_Se_2_ are optimal, with the addition sequence NaN_3_—BAIB—Ph_2_Se_2_.

**Figure 2:**
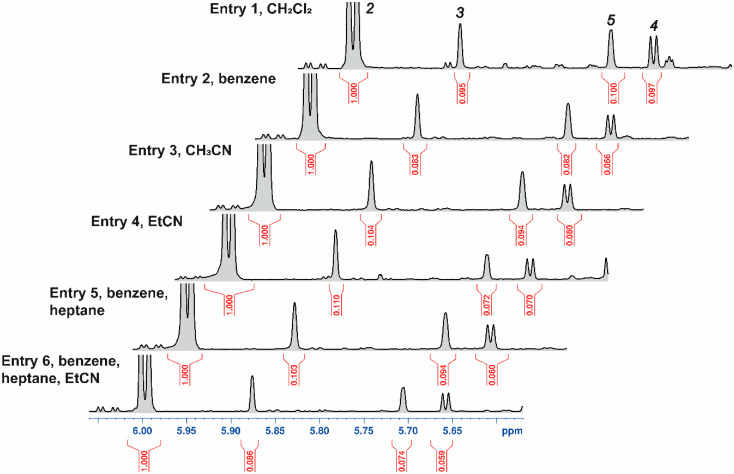
Selected ^1^H NMR spectral region (δ 5.47–6.06 ppm) of the crude reaction mixtures of tri-acetylgalactal **1** APS (Table 1). The H-1 signals of compounds **2**, **3**, **5,** and **4** are indicated by their respective labels. A more detailed spectrum is provided in the Supplementary Information (Figure S1), where the non-identifiable byproducts are shown in gray.

The outcomes of the APS experiments were evaluated by ^1^H NMR analysis of the crude mixtures in CDCl_3_, obtained after the reaction mixtures were quenched with aqueous NaHCO_3_-Na_2_S_2_O_3_, extracted with EtOAc, and concentrated (Figure 2). APS of galactal **1** afforded the target phenyl 2-azido-2-deoxy-1-seleno-D-galactoside **2** together with byproducts **3**–**5**, whose characteristic H-1 signals are shown in Figure 2 (and Figure S1 in Supplementary Information). Talose **3** arises from incomplete stereocontrol, whereas 2-phenylselenoglycosyl azides **4** and **5** originate from the Markovnikov pathway. In addition, a substantial amount of poorly identifiable byproducts was formed. These were evaluated by approximate integration and taken into account when assessing the reaction efficiency (see Figure S1 in the Supplementary Information, where the signals of compounds **2–5** are shown in color and the non-identifiable byproducts in gray). The solvent set included non-polar benzene as well as heptane (to decrease polarity) and nitriles capable of Na^+^ solvation (thus enhancing NaN_3_ transfer), namely MeCN and EtCN. The latter combines the CN group’s solvating ability with lower polarity.

Ratios of APS products derived from galactal **1** and yields of **2**, calculated from the integration of identifiable anomeric signals in the ^1^H NMR spectra, together with reaction times, are summarized in Table 1. Full conversion of galactal **1** in *CH_2_Cl_2_* (Entry 1) required four days and furnished an NMR yield of 77%. The reaction generated copious foam, which separated the solid and liquid phases and required periodic manual agitation. Switching to *benzene* (Entry 2) mitigated foaming and unexpectedly halved the reaction time to two days while increasing the yield to 80%.

In *MeCN*, the reaction reached completion within 21 h (Entry 3). *Propionitrile (EtCN)* delivered the most favorable outcome (Entry 4): full conversion in 15 h, the highest yield of 2-azido-2-deoxy-1-selenogalactose **2**, an improved Gal:Tal ratio, and a superior anti-Markovnikov/Markovnikov ratio. The crude mixture produced in EtCN gave a noticeably cleaner ^1^H-NMR spectrum than those obtained from reactions in CH_2_Cl_2_ or benzene and contained fewer non-identifiable byproducts (see the “gray” signals in Figure S1 in the Supplementary Information). The product also crystallized readily during work-up.

In the benzene/heptane (1:1) mixture (Entry 5), the amount of unidentified byproducts was lower than in neat benzene. To combine the desirable features of the individual solvents, a ternary mixture of *benzene*/*EtCN*/*heptane* (1:1:1, Entry 6) was evaluated. This blend and EtCN afforded the highest NMR yield, optimal C-2 stereoselectivity and regioselectivity, minimal Ph_2_Se_2_ oxidation products, and excellent crystallizability.

**Scheme 2:**
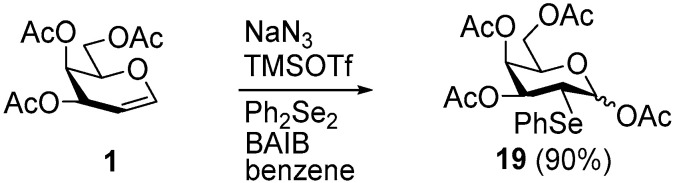
Formation of 2-phenylseleno-2-deoxy-1-*O*-acetate **19** during APS of galactal **1** in benzene with a catalytic amount of TMSOTf (Table 2, Entry 1).

To probe whether catalytic silyl additives could enhance N_3_^−^ transfer and thereby shorten APS reactions, galactal **1** was subjected to the most effective solvent conditions from Table 1 in the presence of TMSCl or TMSOTf at 3 mol % relative to NaN_3_ (Table 2). In benzene (Entry 1), TMSOTf diverted the pathway, furnishing 2-phenylseleno-2-deoxy-1-*O*-acetyl adduct **19** as the major product after two hours (Scheme 2), whereas TMSCl merely slowed conversion and diminished the yield of the target azidoselenide **2** (Entry 2).

This behavior is consistent with two distinct modes of action of TMSOTf depending on the solvent. In EtCN, the higher coordinating ability and polarity favor its role as a phase-transfer promoter for N_3_^−^, accelerating its delivery to BAIB and increasing the rate of PhSeN_3_ formation. In contrast, in benzene. which lacks coordination ability to Na^+^ TMSOTf, more strongly activates BAIB toward ligand exchange with acetate, thereby favoring the generation of PhSeOAc and PhSe·over N_3_·. Introducing TMSCl into the benzene/EtCN/heptane (1:1:1) system lengthened the reaction (Entry 4).

We next examined the APS of tri-*O*-acetyl-glucal **6** [45] in the most promising solvents identified for galactal **1** and monitored both the combined yield of the 2-azido products as well as the diastereomeric ratio of formed *gluco*- (**7**) and *manno*-product (**8**) (Table 3). The preparative synthesis of glucal **6** is described in Supplementary Information.

Unless otherwise noted, reactions were performed at **6** concentration of 0.21 M and with 0.6 equiv. Ph_2_Se_2_. In benzene (Entry 1), the substrate was consumed within 48 h, affording an equimolar mixture of phenylseleno 2-azido-2-deoxy-glucoside **7** and -mannoside **8** in good combined yield. Reducing the concentration of **6** to 0.07 M in the same solvent (Entry 2) slowed the reaction left starting material unconsumed, and shifted the diastereomeric ratio to favor the *manno*-isomer.

A benzene/heptane (1:1) medium (Entry 3) further decreased the rate (conversion remained incomplete after six days), yet, for the first time in the above experiments, the product ratio favored *gluco*-isomer **7**. This indicated that lower-polarity environments may bias stereochemistry toward *gluco*-product formation. In MeCN (Entry 4) and EtCN (Entry 5), the reaction was again sluggish, and the product mixture showed a slight excess of *manno-isomer* **8**.

Overall, solvent polarity and substrate concentration exert competing effects on both reaction rate and diastereocontrol: neat benzene provides the only case of full conversion, whereas mixed aromatic-aliphatic media offer a potential handle for enhancing *gluco*-selectivity that merits further optimization.

### 2.3. Scaling-Up Experiments

The optimized APS conditions were adopted for the preparative synthesis of phenylseleno 2-azido-2-deoxy-galactoside **2** and -glucoside **7**. Taking into account the results from Table 1 and Table 3, EtCN was selected for APS of galactal **1,** while benzene for glucal **6**.

To ensure homogeneous distribution of the reagents, which is difficult to control upon laboratory scale-up, the NaN_3_-containing mixture was thoroughly homogenized by stirring and sonication, after which BAIB and Ph_2_Se_2_ were added in small portions over about 10 min each. During scale-up, we maintained geometric similarity of the reaction vessels, including the ratio of the stir bar size to the diameter of the reaction mixture. Furthermore, we found that the most reproducible results were obtained when the vessel was chosen such that the ratio of the reaction mixture’s diameter to its height was close to 1:1. Under these conditions, APS of galactal **1** on a 1.1 g (4+ mmol) and further scaling was successful. However, when the reaction was run in a standard 250 mL round-bottom flask with a 2 g charge, it remained incomplete, with about 20% of galactal **1** unreacted. Scaling glucal **6** APS revealed another limitation: in benzene, suspending NaN_3_ uniformly throughout the volume became difficult at glucal loads above 1.5 g, suggesting that this may be the practical upper limit of this protocol.

**Scheme 3:**
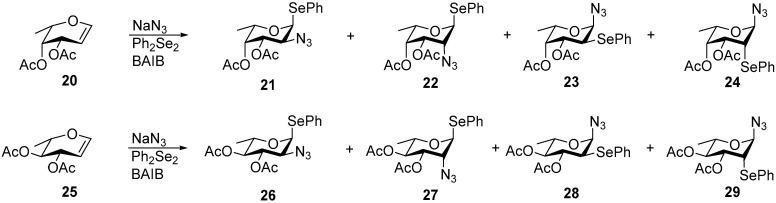
APS of fucal **20** and rhamnal **25** leading to phenyl 2-azido-2-deoxy-1-selenoglycosides **21** and **26**, as well as associated byproducts **22–24**, and **28–29**.

The conditions that proved effective in APS of galactal and glucal at higher loadings were next applied to fucal **20** [46] and rhamnal **25** [47] (Scheme 3, preparative synthesis of **20** and **25** is described in Supplementary information). Solvent choice was guided by analogy: for fucal, whose configuration closely resembles that of galactal, APS was examined in MeCN and EtCN (Table 4, Entries 1 and 2). For rhamnal, on the other hand, the glucal precedent was followed, and APS was evaluated in benzene (Table 4, Entry 3).

APS of fucal **20** (0.5 mmol) in EtCN afforded a combined yield of 65% for the product mixture (Table 4, Entry 1), which included phenylseleno 2-azido-2-deoxyfucoside **21** (58%), accompanied by minor products: phenylseleno 2-azido-2,6-dideoxy-l-taloside **22** (4%), as well as 2-phenylseleno-2,6-dideoxy-l-talosylazide **24**, (2%) and its *fuco*-isomer **23** (1%). These values were calculated from NMR spectra by using the above-described approach after partial separation of the mixture by column chromatography.

When the reaction was conducted in MeCN on a 2.5 mmol scale, the combined yield of the mixture increased to 91%, and the yield of 2-azidofucoside **21** improved markedly to 79%, albeit with a slight decrease in stereoselectivity (Table 4, Entry 2). Although at this stage, pure product **21** could not be isolated, it is known that Zemplén O-deacetylation allows further separation of phenyl 2-azido-2-deoxy-1-phenylseleno-α-l-fucoside by simple crystallization [30].

APS of rhamnal **25** was first carried out using 0.6 equiv of Ph_2_Se_2_, corresponding to the optimized loading established for other glycals in the general APS protocol. Under these conditions, the reaction proceeded slowly, and the conversion remained modest. Increasing the amount of Ph_2_Se_2_ to 1.2 equiv (Table 4, Entry 3) led to marginal improvement: after 40 h, the conversion reached 79%, affording the mixture of **26–29** in 49% isolated yield. NMR analysis of the purified mixture (after removal of the starting material) confirmed the formation of 26% of 2-azidoquinovoside **26**, 19% of 2-azidorhamnoside **27**, and a total of 4% of the phenylseleno byproducts **28** and **29**.

**Scheme 4:**
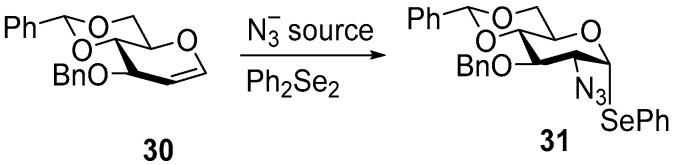
APS of 3-*O*-benzyl-4,6-di-*O*-benzylidene-glucal **30**.

In order to study an alternative approach toward phenylseleno 2-azido-2-deoxy-glucosides, the APS of 3-O-benzyl-4,6-di-O-benzylidene-glucal **30** was studied as the substituent of triacetylglucal **6** (Scheme 4). It was first examined under the heterogeneous conditions that had proved fastest for galactal **1** (EtCN as solvent, a substrate concentration of 0.21 molar, and 0.6 equiv of Ph_2_Se_2_). Synthesis of **30** was carried out through known 2-O-acetyl-3-O-benzyl-4,6-O-benzyliden-β-d-glucopyranoside [48] and is described in Supplementary Information. Under these conditions, only trace amounts of phenylseleno 2-azido-2-deoxy-glucoside **31** were detected sporadically (Table 5, entry 1). Follow-up tests showed that this sporadic activity correlated with traces of μ-*oxo*-bis(acetoxy)iodobenzene (μ-*oxo*-BAIB) [49], a minor impurity occasionally present in commercial BAIB, rather than with the standard oxidant itself.

To clarify the role of μ-*oxo*-BAIB, it was prepared from BAIB (see Supplementary Information) and added (1:1, n/n) to the standard heterogeneous APS of **30** (entry 2). This reaction favored the formation of the *gluco*-product **31**, albeit in only 22% isolated yield. Although not preparatively useful, the striking selectivity encouraged us to revisit the classical homogeneous protocol. With TMSN_3_/BAIB in CH_2_Cl_2_ at −35 °C, the glucoside **31** was obtained in 65% yield after chromatography (entry 3). When BAIB was replaced by BAIB/μ-oxo-BAIB (1:4) at the same temperature, the reaction time shortened from 24 h to 30 min, a 50-fold acceleration, while the isolated yield increased to 76% (entry 4). These results indicate that (i) low temperature is essential for efficient conversion of used glucal **30**, and (ii) μ-*oxo*-BAIB acts as a more selective oxidant than BAIB in this substrate class.

Systematic variation of solvent polarity and phase-transfer conditions revealed that the rate and selectivity of azidophenylselenylation (APS) are governed primarily by the efficiency of N_3_^−^ mass transfer. Improved dispersion of NaN_3_ directly correlated with cleaner reaction profiles and higher yields. For tri-*O*-acetyl-d-galactal **1**, optimal performance in EtCN is attributed to rapid N_3_^−^ delivery and efficient generation of the reactive PhSeN_3_. In contrast, for glucal **6**, benzene afforded the best results, maintaining anti-Markovnikov selectivity through its low polarity.

In EtCN with glucal **6**, the high solubility of NaN_3_ is offset by solvent polarity effects that alter the relative rates of competing pathways, most likely through polarization of the C=C bond and steric differences between glucal **6** and galactal **1**, thereby slowing conversion despite potentially faster mass transfer.

Catalytic TMSCl had little influence on the APS rate or conversion, whereas TMSOTf accelerated galactal APS two- to threefold in EtCN but diverted the reaction toward phenylselenoacetylation (→**19**) in benzene. These observations highlight the dual role of silyl additives as both phase-transfer promoters and electrophilic activators of BAIB, depending on solvent coordination strength.

Scaling experiments confirmed that mass-transfer control remains crucial at preparative levels. APS of galactal **1** in EtCN and glucal **6** in benzene (1.5–2 g, >3 mmol) furnished 76% of crystalline phenylseleno 2-azidogalactoside **2** and 72% of a 1.2:1 mixture of phenylseleno-2-azido-glucose and -mannose isomers **7** and **8**. The same optimized protocol applied to *l*-fucal **20** in MeCN produced a 91% combined yield dominated by 2-azidofucoside **21** (79%), together with minor byproducts **22**–**24**. APS of *l*-rhamnal **25** in benzene gave mainly 2-azidoquinovoside **26** and 2-azidorhamnoside **27** with only traces of phenylseleno derivatives **28** and **29**.

When applied to 3-*O*-benzyl-4,6-*O*-benzylidene-glucal **30**, the use of μ-*oxo*-BAIB markedly promoted formation of phenylseleno-2-azidoglucose; under homogeneous TMSN_3_/CH_2_Cl_2_ conditions, the yield reached 76%, 12–15% higher than with BAIB alone.

Thus, the study provides both a practical synthetic route to biologically relevant azido-selenoglycoside building blocks and mechanistic insight into the factors that govern APS selectivity and scalability.

## 3. Materials and Methods

### 3.1. General Methods

EtCN, benzene, and heptane were used as received. Comparative runs with EtCN (dried by successive distillation over P_2_O_5_ and CaH_2_) and benzene (distilled over sodium/benzophenone ketyl) showed no change in the reaction rate or isolated yield of the APS products. CH_2_Cl_2_, MeCN were purified as described for EtCN and were not tested for their impact on the efficiency of the APS. Analytical thin-layer chromatography (TLC) was performed on silica gel 60 F254 aluminum sheets (Merck, Darmstadt, Germany), and visualization was accomplished using UV light (Analytik Jena (UVP), Jena, Germany) or by charring at 150 °C with 10% (*v*/*v*) H_3_PO_4_ in isopropanol. Column chromatography was performed on silica gel 60, 40–63 μm (Merck) and, when specified on, 25–40 µm (Merck). Optical rotation values were measured using a JASCO DIP-360 polarimeter (JASCO, Tokyo, Japan) at a specified temperature in the specified solvent. ^1^H and ^13^C NMR spectra were recorded at 300, 400, and 600 MHz as detailed for each compound. Chemical shifts were referenced to residual solvent signals. Structural assignments were made with additional information from gCOSY, gHSQC. High-resolution mass spectra were acquired by electrospray ionization on a MicrO TOF II (Bruker Daltonics, Billerica, MA, USA) instrument.

### 3.2. Safe Note

A moderate evolution of N_2_ can accompany PhI(N_3_)_2_ side pathways; therefore, vigorous stirring in fully closed vessels should be avoided. For safety reasons, EtOAc is recommended for the aqueous work-up instead of CHCl_3_, which may form unstable species under azidation conditions.

### 3.3. Representative Example of Heterogeneous APS Test Reaction Protocol

*3,4,6-tri-O-acetyl-2-azido-2-deoxy-1-(phenylseleno)-α-d-galactopyranoside (**2**) (Table 1, Entry 4)*

To the tri-*O*-acetylgalactal **1** (0.42 mmol, 0.21 M) solution in EtCN (2 mL) cooled to +15 °C, NaN_3_ (54.6 mg, 0.84 mmol) was first added, followed by BAIB (162.3 mg, 0.504 mmol). After 20 min of stirring, Ph_2_Se_2_ (78.7 mg, 0.25 mmol) was introduced into the reaction mixture. The progress of the reaction was monitored by TLC (toluene/EtOAc, 7:1). Once the reaction was complete, the contents of the test tube were diluted 4–5 times with EtOAc and transferred to a separatory funnel containing an aqueous mixture of 10% Na_2_S_2_O_3_ and saturated NaHCO_3_ (1:1). The funnel was shaken, and after separation of the organic phase, the aqueous phase was washed three additional times with EtOAc. The combined organic extracts were concentrated under reduced pressure, and the dry residue was analyzed by NMR. Data for phenylseleno 2-azido-2-deoxy-galactoside (**2**): colorless oil; R*_f_* = 0.40 (toluene/EtOAc, 7:1). ^1^H NMR (400 MHz, CDCl_3_): δ 7.63–7.56 (m, 2H, PhSe), 7.32–7.24 (m, 3H, PhSe), 5.99 (d, *J*_1,2_ = 5.4 Hz, 1H, H-1), 5.46 (br d, *J*_4,3_ = 3.3 Hz, 1H, H-4), 5.11 (dd, *J*_3,2_ = 10.8 Hz, *J*_3,4_ = 3.2 Hz, 1H, H-3), 4.25 (dd, *J*_2,1_ = 5.4 Hz, *J*_2,3_ = 10.9 Hz, 1H, H-2), 4.66 (br t, *J*_5,6_ = 6.5 Hz, 1H, H-5), 4.04 (m, 2H, H-6), 2.14, 2.05, 1.96 (3s, 3×1H, CH_3_ (Ac)). ^13^C{^1^H } NMR (100.6 MHz, CDCl_3_): δ 170.5, 170.1, 169.7 (C=O (Ac)), 134.9 (*ipso* Ar(PhSe)), 129.4, 128.3 (PhSe), 81.2 (C-1), 71.4 (C-3), 69.1 (C-5), 67.3 (C-4), 61.7 (C-6), 20.8 (CH_3_(Ac)). Data are consistent with the literature [37]. NMR data for products **3–5**, identified as components of the mixture **2–5** obtained after APS of galactal, are provided in the Supplementary Information and are consistent with the reported literature data [37].

### 3.4. Representative Example of Heterogeneous APS Test Reaction Protocol with Additive

*3,4,6-tri-O-acetyl-2-azido-2-deoxy-1-(phenylseleno)-α-d-galactopyranoside (**2**) (Table 2, Entry 3)*

The reaction was conducted as described above, except that TMSOTf (6.3 µL, 4 mol % relative to NaN_3_) was added immediately after the addition of Ph_2_Se_2_.

### 3.5. General Large-Scale APS Procedure

*3,4,6-tri-O-acetyl-2-azido-2-deoxy-1-(phenylseleno)-α-d-galactopyranoside (**2**)*

To a solution of tri-*O*-acetylgalactal **1** (1.10 g, 4.0 mmol) in EtCN (20.3 mL), NaN_3_ (523 mg, 8.05 mmol) was added at room temperature under argon in a cylindrical round-bottomed flask (30 mm diameter) equipped with a 23 × 9 mm PTFE-coated stir bar. The mixture was sonicated for 3 min, then stirred for an additional 10 min. After cooling to +15 °C, BAIB (1.53 g, 4.8 mmol) was added portion-wise over 10 min, and stirring was continued for 15 min until the reaction mixture developed a pearlescent sheen. Ph_2_Se_2_ (750.6 mg, 2.4 mmol) was then added portion-wise over 15 min, and the reaction was stirred at 1000–1100 rpm for 27 h, until completion. The mixture was quenched as described for the representative small-scale test reaction. After quenching and concentration, the solids (2.3 g) were purified by crystallization from *i*PrOH (3.8 mL) to afford pure phenyl seleno-2-azido-galactoside **2** (1.43 g, 76%).

*3,4,6-tri-O-acetyl-2-azido-2-deoxy-1-(phenylseleno)-α-l-gluco- (**7**) and mannopyranoside (**8**)*

APS of glucal **6** was carried out according to the general large-scale procedure described for galactal **1**, except that benzene was used as the solvent. The reaction afforded a combined yield of 70–72% for the isolated mixture, consisting of **7**, **8**, and minor byproducts **9** and **10** in a 1.2:1:0.2:0.1 ratio, as determined by NMR after partial separation by column chromatography on SiO_2_ (toluene-EtOAc gradient, 10 → 35%). NMR data for products **7**–**10** from the APS of: ^1^H NMR (600 MHz, CDCl_3_): δ 7.59–7.52 (m, 5.8H, Ph), 7.34–7.26 (m, 8.8H, Ph), 5.92 (d, *J*_1,2_ = 5.6Hz, 1H, H-1**^7^**), 5.78 (d, *J*_1,2_ = 1.4 Hz, 1.3H, H-1**^8^**), 5.65 (d, *J*_1,2_ = 1.8 Hz, 0.24H, H-1**^9^**), 5.40 (t, 0.24H, H-4**^9^**), 5.36 (t, *J*_4,3_ = *J*_4,5_ = 9.7 Hz, 1.3H, H-4**^8^**), 5.31–5.24 (m, 2.6H, H-3**^7,8^**), 5.04 (dd, *J* = 9.2 Hz, *J* = 10.2 Hz, H-4**^7^**), 4.92 (m, 1H, H-5**^7^**), 4.40 (m, 1.3H, H-5**^8^**), 3.35 (dd, *J*_2,1_ = 1.4 Hz, *J*_2,3_ = 3.7 Hz, 1.3H, H-2**^8^**), 4.29–4.25 (m, 2.3H, H-6A**^7,8^**), 4.09–4.03 (m, 2.3H, H-6B**^8^**, H-2**^7^**), 3.94 (dd, *J*_6B,5_ = 2.2 Hz, *J*_6B,6A_ = 12.5 Hz, 1H, H-6B**^7^**), 3.80 (dd, *J*_2,1_ = 1.9 Hz, *J*_2,3_ = 4.4 Hz, H-2**^9^**), 2.15, 2.12, 2.08, 2.06, 2.05, 2.04, 2.00 (7×s, 28.6H, CH_3_(Ac)). ^13^C{^1^H } NMR (150.9 MHz, CDCl_3_): δ 170.7, 170.6, 170.1, 169.9, 169.6 (C=O (Ac)), 134.7, 134.3, 129.6, 129.4, 128.6, 128.4 (PhSe), 90.3 (C-1**^9^**), 83.8 (C-1**^7^**), 82.7 (C-1**^8^**), 74.0 (C-5**^9^**), 73.0 (C-3**^7^**), 71.7 (C-3**^8^**), 71.4 (C-5**^8^**), 70.2 (C-5**^7^**), 68.5 (C-4**^7^**), 66.7 (C-4**^9^**), 66.0 (C-4**^8^**), 63.5 (C-2**^8^**), 62.4 (C-2**^7^**), 62.3 (C-6**^9^**), 62.2 (C-6**^8^**), 61.9 (C-6**^7^**), 47.2 (C-2**^9^**), 20.8 (CH_3_ (Ac)) and are consistent with the literature [35].

*3,4-di-O-acetyl-2-azido-2-deoxy-1-(phenylseleno)-α-l-fucopyranoside (**21**)*

APS of fucal **20** was carried out according to the general large-scale procedure described for galactal **1**, except that MeCN was used as the solvent. The reaction afforded a combined yield of 91% for the isolated mixture, consisting of **21** (79%), phenyl 2-azido-2,6-dideoxy-l-taloside **22** (8%), 2-phenylseleno-2,6-dideoxy-l-talosyl acetate **24** (2%), and 2-deoxy-2-phenylseleno-fucosyl acetate **23** (1%), as determined by NMR after partial separation by column chromatography on SiO_2_ (petroleum ether-EtOAc gradient, 1:0 → 9:1). Data for compounds **21**–**24**: ^1^H NMR (300 MHz, CDCl_3_): δ 7.63–7.54 (m, 3H), 7.37–7.26 (m, 5H), 5.97 (d, *J* = 5.4 Hz, 1H, H-1**^21^**), 5.86 (br. s, 0.3H, H-1**^22^**), 5.70 (s, 0.13H, H-1**^24^**), 5.65 (d, *J* = 3.8 Hz, 0.04H, H-1**^23^**), 5.40–5.26 (m, 2H, H-3, H-4), 5.15 (dd, *J* = 10.8, 3.2 Hz, 1H, H-3**^21^**), 4.52 (m, 1.3H, H-5**^21^**^,**22**^), 4.36–4.21 (m, 1.2H, H-5**^24^**^,**23**^), H-2**^21^**) 4.10–4.05 (m, 0.3H, H-2**^22^**), 3.62–3.55 (m, 0.04H, H-2**^23^**), 3.49–3.44 (m, 0.13H, H-2**^24^**), 2.19 (s, 3H), 2.09 (s, 3H), 1.31–1.17 (m, 2H, H-6), 1.11 (d, *J* = 6.5 Hz, 3H, H-6**^21^**). ^13^C NMR (75.5 MHz, CDCl_3_): δ 170.3, 169.7, 134.6, 134.2, 133.9, 129.5, 129.4, 129.1, 128.2, 128.0, 92.0 (C-1**^24^**), 91.1 (C-1**^23^**), 84.4 (C-1**^21^**), 83.3 (C-1**^22^**), 71.6 (C-3**^21^**), 70.6 (C-3**^23^**), 70.1 (C-4**^21^**), 69.0, 68.6 (C-3**^22^**), 68.3, 67.4, 67.3, 67.1, 59.5 (C-2**^22^**), 58.7 (C-2**^21^**), 45.7 (C-2**^24^**), 43.5 (C-2**^23^**), 30.0, 29.7, 20.9, 20.6, 20.6, 16.3 (C-6), 16.0 (C-6), 15.8 (C-6). The characterization data for **21** are consistent with those reported [50].

*3,4-di-O-acetyl-2-azido-2-deoxy-1-(phenylseleno)-α-l-quinovoso- (**26**) and -rhamnopyranosides (**27**)*

APS of rhamnal **25** was carried out according to the general large-scale procedure described for galactal **1**, except that benzene was used as the solvent and 1.2 equiv Ph_2_Se_2_ was employed. The reaction afforded a combined yield of 49% for the isolated mixture, consisting of **26** (26%), phenyl 2-azido-2,6-dideoxy-l-rhamnoside **27** (19%), 2-phenylseleno-2,6-dideoxy-l-quinovosyl acetate **28** and 2-deoxy-2-phenylseleno-rhamnosyl acetate **29** (4%), as determined by NMR after partial separation by column chromatography on SiO_2_ (toluene-EtOAc gradient, 1:0 → 98:2). Data for compounds **26**–**29**: ^1^H NMR (300 MHz, CDCl_3_) δ 7.73–7.53 (m, 4H), 7.46–7.13 (m, 7H), 5.85 (d, *J*_1,2_ = 5.4 Hz, 1H, H-1**^26^**), 5.70 (d, *J*_1,2_ = 1.2 Hz, 0.7H, H-1**^27^**), 5.62–5.56 (m, 0.15H, H-1**^28^**^,**29**^), 5.37–5.10 (m, 2.7H, H-3, H-4), 4.80 (t, *J* = 9.6 Hz, 1H, H-4**^26^**), 4.39–4.16 (m, 2.4H, H-2**^27^**, H-5**^26^**, H-5**^27^**), 4.07–3.97 (m, 1H, H-2**^26^**), 3.83 (dd, *J* = 4.0, 1.7 Hz, 0.1H, H-2**^29^**), 2.13–2.04 (m, 11H, Ac), 1.29–1.20 (m, 3H, H-6**^27^**, H-6**^28^**^,**29**^, 1.15 (d, *J* = 6.2 Hz, 3H, H-6**^26^**). ^13^C NMR (76 MHz, CDCl_3_) δ 134.6, 134.1, 129.4, 129.2, 128.3, 128.1, 83.9 (C-1**^26^**), 82.6 (C-1**^27^**), 73.6 (C-4**^26^**), 72.9 (C-3**^26^**), 71.5, 70.8, 69.7 (C-5**^27^**), 68.4 (C-5**^26^**), 63.8, 62.7 (C-2**^26^**), 20.7, 20.6, 17.2 (C-6), 17.0 (C-6). The characterization data for **26** and **27** are consistent with those reported [35].

### 3.6. Representative Example of the APS of Benzylidene-Protected Glucal 30 Using μ-Oxo-bis(acetyloxy)iodobenzene as the Oxidant

*Phenyl 2-azido-3-O-benzyl-4,6-O-benzylidene-2-deoxy-1-selenyl-α-d-glucopyranoside (**31**) (Table 4, Entry 4)*

A solution of glucal **30** (40.0 mg, 0.12 mmol) and Ph_2_Se_2_ (38.4 mg, 0.12 mmol) in dry CH_2_Cl_2_ (1.4 mL) was cooled to −35 °C under argon. While stirring, TMSN_3_ (0.24 mmol, 32.4 µL) was added, followed by μ-*oxo*-BAIB and BAIB mixture (73 mg, containing 80 mol % μ-*oxo*-BAIB and 20 mol % BAIB). After 30 min, TLC analysis showed complete consumption of the starting material. The reaction mixture was then diluted with CH_2_Cl_2_ and washed with saturated aqueous NaHCO_3_. The aqueous phase was extracted twice with CH_2_Cl_2_, and the combined organic extracts were dried over Na_2_SO_4_, filtered, and concentrated under reduced pressure. Purification by silica-gel chromatography (petroleum ether-EtOAc gradient, 40:1 → 15:1) afforded colorless crystals of phenyl 2-azido-3-*O*-benzyl-4,6-*O*-benzylidene-2-deoxy-1-seleno-α-d-glucopyranoside **31** (49 mg, 76%). Data for **31**: ^1^H NMR (CDCl_3_,300 MHz): δ 7.72 (dd, *J* = 7.8, 1.7 Hz, 1H), 7.63 (dd, *J* = 8.0, 1.6 Hz, 4H), 7.60–7.21 (m, 32H), 5.89 (d, *J*_1,2_ = 9.5 Hz, 1H, H-1), 5.62 (s, 1H, PhC*H*), 5.01 (d, *J*_AB_ = 10.9 Hz, 1H, BnA), 4.81 (d, *J*_AB_ = 10.9 Hz, 1H, BnB), 4.37 (dd, *J*_6A,6B_ = 10.5, *J*_6A,5_ = 5.0 Hz, 1H, H-6A), 3.86–3.72 (m, 2H, H-4, H-6B), 3.64 (dd, *J*_3,2_ = 10.6, *J*_3,4_ = 8.7 Hz, 1H, H-3), 3.50 (td, *J*_5,4_ = 9.7, *J*_5,6A_ = 5.0 Hz, 1H, H-5), 3.30 (dd, *J*_2,3_ = 10.6, *J*_2,1_ = 9.5 Hz, 1H, H-2), 1.99 (s, 3H, Ac). ^13^C{^1^H} NMR (76 MHz, CDCl_3_): δ (ppm) 168.9, 138.1, 137.1, 135.9, 135.3, 129.3, 129.2, 129.1, 129.0, 128.5, 128.4, 128.3, 128.3, 128.3, 128.2, 127.9, 127.7, 127.1, 126.1, 126.0, 101.3 (Ph*C*H), 94.4 (C-1), 83.1 (C-4), 78.0 (C-3), 77.5, 77.5, 76.6, 75.3 (Bn), 68.5 (C-6), 66.5 (C-5), 48.7 (C-2), 20.7 (Ac). Characterization data are consistent with those reported [51].

## 4. Conclusions

It was shown that APS efficiency critically depends on controlling N_3_^−^ mass transfer, which can be regulated by solvent polarity. While the reaction in general benefits from a compromise between solvent polarity, which promotes N_3_^−^ mass transfer, and unpolar media, which favor the desired regioselectivity, glycal substrates with different C-4 configurations (*galacto*- and *gluco*-series) require distinct solvent types to achieve optimal conversion and purity. The optimized heterogeneous APS protocol developed in this study provides reproducible, gram-scale access to three valuable phenylseleno 2-azido-2-deoxy-monosaccharides, namely galactoside **2**, glucoside **7**, and fucoside **21**, and extends the methodology to quinovose **26** and rhamnose **27** derivatives. Moreover, application of μ-*oxo*-BAIB to the APS of 3-O-benzyl-4,6-O-benzylidene-*d*-glucal **30** enabled isolation of a pure *gluco*-configured product **31** in high yield, underscoring the potential of this oxidant for selective and scalable synthesis. Further study of the discovered protocol and its dependence on glycal structure is under investigation in this laboratory and will be reported in due course.

## Figures and Tables

**Table 1:** The results APS of galactal **1** (Scheme 1) at full conversion: yields of target anti-Markovnikov’s *galacto*-adduct **2** and its ratios with *talo*-isomer **3**, as well as the ratios of target anti-Markovnikov’s (**2**, **3**) and Markovnikov’s adducts **4** and **5** with *galacto*- and *talo*-configurations.

Entry	Solvent	Time, h	Yield of 2, % ^1^	2:3	(2 + 3):(4 + 5)
1	CH_2_Cl_2_	96	77	10.6:1	5.5:1
2	C_6_H_6_	48	80	9.1:1	7.8:1
3	MeCN	21	78	9.7:1	6.3:1
4	EtCN	15	81	12.1:1	7.3:1
5	C_6_H_6_/heptane (1:1)	48	78	9.7:1	6.4:1
6	C_6_H_6_/EtCN/heptane (1:1:1)	48	82	11.7:1	8.1:1

^1^ The yield of **2** was calculated as Y**_2_** = (100 × I**_2_**)/(I**_1_** + I**_2_** + I**_3_** + I**_4_** + I**_5_**), where I**_1_**–I**_5_** are the integrals of the H-1 signals of compounds **1–5**, respectively, in the ^1^H-NMR-spectra of the crude mixtures (Figure 1). In addition, the fractions of compounds **3–5**, used to determine the **2**:**3** and (**2** + **3**):(**4** + **5**) ratios, which were calculated using the same formula as the yield of product **2**, Y**_X_** = (100 × I**_X_**)/(I**_1_** + I**_2_** + I**_3_** + I**_4_** + I**_5_**).

**Table 2:** The results of APS of galactal **1** in the presence of TMS-additives (3 mol %) (Scheme 1).

Entry	Solvent	TMS- Additive	Time, h	Major Product	Yield, %
1	C_6_H_6_	TMSOTf	2	**19**	70
2	C_6_H_6_	TMSCl	72	**2**	72
3	EtCN	TMSOTf	4.5 h	**2**	80
4	C_6_H_6_/EtCN/heptane (1:1:1)	TMSCl	144	**2**	90

**Table 3:** Solvent effect on the APS of tri-*O*-acetyl-glucal **6** and formation of *gluco*- and *manno*-product **7** and **8** (Scheme 1) ^1^.

Entry	Solvent	Conversion, % ^1^	Time, d	Total yield of 7 and 8, % ^2^	Yield of 7, %	7:8
1	C_6_H_6_	100	2	100	52	1:1
2	C_6_H_6_ ^3^	73	5	73	29	1:1.5
3	C_6_H_6_:heptane (1:1)	66	6	58	33	1.7:1
4	MeCN	81	6	81	36	1:1.3
5	EtCN	85	6	83	37	1:1.2

^1^ The conversion of **6** was expressed as 100 × (1 − I**_6_**)/(I**_6_** + I**_7_** + I**_8_** + I**_9_**+I**_10_**), where I**_6_**–I**_10_** are the integrals of the H-1 signals of compounds **6–10**, respectively, in the ^1^H-NMR-spectra of crude mixtures. ^2^ Analogously, yields of **7** and **8** were calculated as: Y**_X_** = (100 × I**_X_**)/(I**_6_** + I**_7_** + I**_8_** + I**_9_** + I**_10_**). ^3^ All reactions were conducted at a substrate concentration of 0.21 M with 0.6 equiv Ph_2_Se_2_, except Entry 2, where the substrate **6** concentration was 0.07 M.

**Table 4:** Results of the APS of fucal **20** and rhamnal **25** (Scheme 3): yields of product mixtures **21–24** (Entries 1, 2) and **26**–**29** (Entry 3), and the ratios of the corresponding 2-azido-stereoisomers: *fuco*- and *talo*-adducts **21** and **22** (Entries 1–2), *quino*- and *rhamno*-adducts **26** and **27** (Entry 3), as well as the ratios of anti-Markovnikov (**21** + **22**) and (**26** + **27**) to the corresponding Markovnikov adducts (**23** + **24**) and (**28** + **29**) adducts.

Entry	Glycal, mmol	Solvent	Products	Total Yield, % ^1^	Product Ratios ^2^	
1	**20**, 0.5	EtCN	**21**, **22**, **23**, **24**	65	15:1 (**21**:**22**)	21:1 (**21**+**22**:**23**+**24**)
2	**20**, 2.5	MeCN	**21**, **22**, **23**, **24**	91	9:1 (**21**:**22**)	29:1 (**21**+**22**:**23**+**24**)
3	**25**, 0.5	C_6_H_6_	**26**, **27**, **28**, **29**	49 ^3^	1.4:1 (**26**:**27**)	11:1 (**26**+**27**:**28**+**29**)

^1^ Isolated yield of mixture of 2-azido-epimers and 2-phenylselenoisomers. ^2^ Ratios determined by ^1^H NMR; calculated from H-1 integrals as Y**_X_** = (100 × I**_X_**)/∑ I**_i_** where X denotes one of the products (**21**–**24** for Entries 1, 2 or **26**–**29** for Entry 3), and I_i_ are the integrals of the H-1 signals of all products in the corresponding products mixture. ^3^ 1.2 equiv Ph_2_Se_2_ was required to achieve the best conversion of the starting material.

**Table 5:** Influence of oxidant and solvent on the APS of glucal **30** (Scheme 4).

Entry	Solvent	Oxidant ^1^	N_3_^−^ Source	T, °C ^2^	Yield 31, %
1	EtCN	BAIB	NaN_3_	−20 → 23	traces
2	EtCN	BAIB/µ*O*-BAIB (1:1)	NaN_3_	−20 → 10	22
3	CH_2_Cl_2_	BAIB ^3^	TMSN_3_	−25	65
4	CH_2_Cl_2_	BAIB/µ*O*-BAIB ^3^ (1:4)	TMSN_3_	−35	76

^1^ µ*O*-BAIB = μ-*oxo*-bis(acetoxy)iodobenzene. ^2^ Initial temperature; arrows denote gradual warming to the final value shown. All reactions employed 0.6 equiv of Ph_2_Se_2_ at a substrate concentration of 0.21 M. ^3^ The total reaction time was 24 h when using BAIB alone (Entry 3), and 30 min with the BAIB/μ-*oxo*-BAIB mixture (Entry 4).

## Data Availability

The original contributions presented in this study are included in the article and Supplementary Information. Further inquiries can be directed to the corresponding author.

## Supplementary Materials

The following Supplementary information can be downloaded at: https://www.mdpi.com/article/10.3390/molecules31010054/s1. It contains full experimental procedures fot the preparation of glycals **1**, **6**, **20**, **25**, and **30**, the synthesis of µ-*oxo*-BAIB, and additional optimization experiments including: Scheme S1. Preparation of glycals **1** and **6**; Scheme S2. Preparation of l-fucal **20**; Scheme S3. Preparation of l-rhamnal **25**; Scheme S4. Preparation of glucal **30**; Scheme S5. Synthesis of µ-*oxo*-BAIB. Complete NMR data for compounds **1**–**9** and **16** are provided, together with the ^1^H NMR spectra of crude APS mixtures (Table 1, Table 2, Table 3 and Table S1): Figure S1. Comparison of ^1^H NMR spectra of crude mixtures of APS transformation of galactal **1** in various solvents (Table 1) recorded in CDCl_3_; Figure S2. Selected ^1^H NMR spectral region (δ 5.47–6.06 ppm) of the crude reaction mixtures of tri-acetylgalactal **1** APS (Table S1); Figure S3. Selected ^1^H NMR spectral region (δ 5.47–6.06 ppm) of the crude reaction mixtures of tri-acetylgalactal **1** APS (Table 2); Figure S4. Selected ^1^H NMR spectral region (δ 5.47–6.06 ppm) of the crude reaction mixtures tri-acetylglucal **6** APS (Table 3). Tables S1–S6 summarize the reaction conditions, reagent quantities, and NMR assignments: Table S1. Selected APS of galactal 1 (in addition to Table 1, Scheme 1) and outcome determined by NMR; Table S2. Reagent quantities, solvent system, and substrate concentration for azidophenylselenylations of galactal **1** presented in Table 1 and Table 2; Table S3. Reagent quantities, solvent system, and substrate concentration for azidophenylselenylations of glucal 6 presented in Table 3; Table S4. Reagent quantities, solvent system, and substrate concentration for azidophenylselenylations of galactal **1** presented in Table S1; Table S5. ^1^H-NMR data for compounds **1**–**9**, **16** (CDCl_3_); Table S6. ^13^C{^1^H}-NMR data for compounds **1**–**9**, **16** (CDCl_3_).

## Abbreviations

The following abbreviations are used in this manuscript:

APS	Azidophenylselenylation
BAIB	[Bis(acetoxy)iodo]benzene
EtCN	Propionitrile
MeCN	Acetonitrile
µ-*oxo*-BAIB	μ-*oxo*-bis(acetoxy)iodobenzene (BAIB dimer)

## References

[1] Østerlid K.E., Li S., Unione L., Bino L.D., Sorieul C., Carboni F., Berni F., Bertuzzi S., Van Puffelen B., Arda A. (2025). Synthesis, Conformal Analysis, and Antibody Binding of *Staphylococcus aureus* Capsular Polysaccharide Type 5 Oligosaccharides. Angew. Chem..

[2] Geng X., Wang Y., Zhang P., Zhang H., Xu L., Wang H., Gao J. (2025). Chemical Synthesis of Conjugation-Ready Oligosaccharide Haptens of *Pseudomonas aeruginasa* O11 and *Staphylococcus aureus* Type 5. Org. Lett..

[3] Bhaumik I., Misra A.K. (2017). Convergent Synthesis of the Tetrasaccharide Repeating Unit of the *O* -Polysaccharide of *Salmonella enterica* O41. ChemistrySelect.

[4] Kumar H., Mandal P.K. (2019). Synthetic Routes Toward Pentasaccharide Repeating Unit Corresponding to the O-Antigen of *Escherichia coli* O181. Tetrahedron Lett..

[5] Das R., Mukhopadhyay B. (2013). Chemical Synthesis of the Tetrasaccharide Repeating Unit of the O-Antigenic Polysaccharide from *Plesiomonas shigelloides* Strain AM36565. Carbohydr. Res..

[6] Zhang H., Wang X., Meng Y., Yang X., Zhao Q., Gao J. (2022). Total Synthesis of the Tetrasaccharide Haptens of *Vibrio vulnificus* MO6-24 and BO62316 and Immunological Evaluation of Their Protein Conjugates. JACS Au.

[7] Paul A., Kulkarni S.S. (2023). Total Synthesis of the Repeating Units of *Proteus penneri* 26 and *Proteus vulgaris* TG155 via a Common Disaccharide. Org. Lett..

[8] Lemieux R.U., Ratcliffe R.M. (1979). The Azidonitration of Tri-*O*-Acetyl-d-Galactal. Can. J. Chem..

[9] Plattner C., Höfener M., Sewald N. (2011). One-Pot Azidochlorination of Glycals. Org. Lett..

[10] Liu S.-X., Tsai Y.-T., Lin Y.-T., Li J.-Y., Chang C.-C. (2019). Design and Synthesis of Trivalent Tn Glycoconjugate Polymers by Nitroxide-Mediated Polymerization. Tetrahedron.

[11] Araki K., Kawa M., Saito Y., Hashimoto H., Yoshimura J. (1986). Synthetic Studies on Glycocinnamoylspermidine. II. Synthesis of 4-*O*-(2-Azido-2-Deoxy-d-Xylopyranosyl)-2-Azido-2-Deoxy-d-Xylopyranose Derivatives. Bull. Chem. Soc. Jpn..

[12] Ali S., Hendriks A., van Dalen R., Bruyning T., Meeuwenoord N., Overkleeft H.S., Filippov D.V., van der Marel G.A., van Sorge N.M., Codée J.D.C. (2021). (Automated) Synthesis of Well-Defined *Staphylococcus aureus* Wall Teichoic Acid Fragments. Chem.—A Eur. J..

[13] Wang L., Zhang Y., Overkleeft H.S., van der Marel G.A., Codée J.D.C. (2020). Reagent Controlled Glycosylations for the Assembly of Well-Defined Pel Oligosaccharides. J. Org. Chem..

[14] Shou K., Zhang Y., Ji Y., Liu B., Zhou Q., Tan Q., Li F., Wang X., Lu G., Xiao G. (2024). Highly Stereoselective α-Glycosylation with GalN_3_ Donors Enabled Collective Synthesis of Mucin-Related Tumor Associated Carbohydrate Antigens. Chem. Sci..

[15] Slättegård R., Teodorovic P., Kinfe H.H., Ravenscroft N., Gammon D.W., Oscarson S. (2005). Synthesis of Structures Corresponding to the Capsular Polysaccharide of *Neisseria meningitidis* Group A. Org. Biomol. Chem..

[16] van der Vorm S., Overkleeft H.S., van der Marel G.A., Codée J.D.C. (2017). Stereoselectivity of Conformationally Restricted Glucosazide Donors. J. Org. Chem..

[17] Lourenço E.C., Ventura M.R. (2016). Improvement of the Stereoselectivity of the Glycosylation Reaction with 2-Azido-2-Deoxy-1-Thioglucoside Donors. Carbohydr. Res..

[18] Bhetuwal B.R., Wu F., Meng S., Zhu J. (2020). Stereoselective Synthesis of 2-Azido-2-Deoxy-β-d-Mannosides via Cs_2_CO_3_-Mediated Anomeric O-Alkylation with Primary Triflates: Synthesis of a Tetrasaccharide Fragment of *Micrococcus luteus* Teichuronic Acid. J. Org. Chem..

[19] Mironov Y.V., Sherman A.A., Nifantiev N.E. (2004). Homogeneous Azidophenylselenylation of Glycals Using TMSN_3_–Ph_2_Se_2_–PhI(OAc)_2_. Tetrahedron Lett..

[20] Khatuntseva E.A., Sherman A.A., Tsvetkov Y.E., Nifantiev N.E. (2016). Phenyl 2-Azido-2-Deoxy-1-Selenogalactosides: A Single Type of Glycosyl Donor for the Highly Stereoselective Synthesis of α- and β-2-Azido-2-Deoxy-d-Galactopyranosides. Tetrahedron Lett..

[21] Kazakova E.D., Yashunsky D.V., Krylov V.B., Bouchara J.-P., Cornet M., Valsecchi I., Fontaine T., Latgé J.-P., Nifantiev N.E. (2020). Biotinylated Oligo-α-(1→4)-d-Galactosamines and Their N-Acetylated Derivatives: α-Stereoselective Synthesis and Immunology Application. J. Am. Chem. Soc..

[22] Kazakova E.D., Yashunsky D.V., Nifantiev N.E. (2021). The Synthesis of Blood Group Antigenic A Trisaccharide and Its Biotinylated Derivative. Molecules.

[23] Kärkkäinen T.S., Ravindranathan Kartha K.P., MacMillan D., Field R.A. (2008). Iodine-Mediated Glycosylation En Route to Mucin-Related Glyco-Aminoacids and Glycopeptides. Carbohydr. Res..

[24] Hagen B., van Dijk J.H.M., Zhang Q., Overkleeft H.S., van der Marel G.A., Codée J.D.C. (2017). Synthesis of the *Staphylococcus aureus* Strain M Capsular Polysaccharide Repeating Unit. Org. Lett..

[25] Bedini E., Cirillo L., Parrilli M. (2012). Synthesis of the Trisaccharide Outer Core Fragment of *Burkholderia cepacia* pv. *Vietnamiensis* Lipooligosaccharide. Carbohydr. Res..

[26] Schumann B., Parameswarappa S.G., Lisboa M.P., Kottari N., Guidetti F., Pereira C.L., Seeberger P.H. (2016). Nucleophile-Directed Stereocontrol Over Glycosylations Using Geminal-Difluorinated Nucleophiles. Angew. Chem. Int. Ed..

[27] Li D., Wang J., Wang X., Qiao Z., Wang L., Wang P., Song N., Li M. (2023). β-Glycosylations with 2-Deoxy-2-(2,4-Dinitrobenzenesulfonyl)-Amino-Glucosyl/Galactosyl Selenoglycosides: Assembly of Partially *N*-Acetylated β-(1→6)-Oligoglucosaminosides. J. Org. Chem..

[28] Njeri D.K., Ragains J.R. (2022). Total Synthesis of an All-1,2-*Cis*-Linked Repeating Unit from the *Acinetobacter baumannii* D78 Capsular Polysaccharide. Org. Lett..

[29] Li Z., Shen W., Cao C., Wang Z., Zhang Y., Xue W. (2024). Thiourea-Cu(OTf)_2_/NIS-Synergistically Promoted Stereoselective Glycoside Formation with 2-Azidoselenoglycosides or Thioglycosides as Donors. Org. Biomol. Chem..

[30] Hagen B., Ali S., Overkleeft H.S., van der Marel G.A., Codée J.D.C. (2017). Mapping the Reactivity and Selectivity of 2-Azidofucosyl Donors for the Assembly of *N*-Acetylfucosamine-Containing Bacterial Oligosaccharides. J. Org. Chem..

[31] Linker T. (2020). Addition of Heteroatom Radicals to Endo-Glycals. Chemistry.

[32] Tingoli M., Tiecco M., Chianelli D., Balducci R., Temperini A. (1991). Novel Azido-Phenylselenenylation of Double Bonds. Evidence for a Free-Radical Process. J. Org. Chem..

[33] Czernecki S., Ayadi E., Randriamandimby D. (1994). Seleno Glycosides. 2. Synthesis of Phenyl 2-(N-Acetylamino)- and 2-Azido-2-Deoxy-1-Seleno-α-d-Glycopyranosides via Azido-Phenylselenylation of Diversely Protected Glycals. J. Org. Chem..

[34] Czernecki S., Ayadi E., Randriamandimby D. (1994). New and Efficient Synthesis of Protected 2-Azido-2-Deoxy-Glycopyranoses from the Corresponding Glycal. J. Chem. Soc. Chem. Commun..

[35] Santoyo-Gonzalez F., Calvo-Flores F.G., Garcia-Mendoza P., Hernandez-Mateo F., Isac-Garcia J., Robles-Diaz R. (1993). Synthesis of Phenyl 2-Azido-2-Deoxy-1-Selenoglycosides from Glycals. J. Org. Chem..

[36] Mironov Y.V., Grachev A.A., Lalov A.V., Sherman A.A., Egorov M.P., Nifantiev N.E. (2009). A Study of the Mechanism of the Azidophenylselenylation of Glycals. Russ. Chem. Bull..

[37] Guberman M., Pieber B., Seeberger P.H. (2019). Safe and Scalable Continuous Flow Azidophenylselenylation of Galactal to Prepare Galactosamine Building Blocks. Org. Process Res. Dev..

[38] Fomitskaya P.A., Argunov D.A., Tsvetkov Y.E., Lalov A.V., Ustyuzhanina N.E., Nifantiev N.E. (2021). Further Investigation of the 2-Azido-Phenylselenylation of Glycals. Eur. J. Org. Chem..

[39] Peturova M., Vitiazeva V., Toman R. (2015). Structural Features of the O-Antigen of *Rickettsia typhi*, the Etiological Agent of Endemic Typhus. Acta Virol..

[40] Atiemo-Obeng V.A., Penney W.R., Armenante P., Paul E.L., Atiemo-Obeng V.A., Kresta S.M. (2003). Solid–Liquid Mixing. Handbook of Industrial Mixing.

[41] Varala R., Seema V., Dubasi N. (2022). Phenyliodine(III)Diacetate (PIDA): Applications in Organic Synthesis. Organics.

[42] Soltanzadeh B., Jaganathan A., Yi Y., Yi H., Staples R.J., Borhan B. (2017). Highly Regio- and Enantioselective Vicinal Dihalogenation of Allyl Amides. J. Am. Chem. Soc..

[43] Roessler A., Rys P. (2001). Wenn die Rührgeschwindigkeit die Produktverteilung bestimmt: Selektivität mischungsmaskierter Reaktionen. Chem. Unserer Zeit.

[44] Chang C., Wu C., Lin M., Liao P., Chang C., Chuang H., Lin S., Lam S., Verma V.P., Hsu C. (2019). Establishment of Guidelines for the Control of Glycosylation Reactions and Intermediates by Quantitative Assessment of Reactivity. Angew. Chem. Int. Ed..

[45] Koseki Y., Watanabe T., Kamishima T., Kwon E., Kasai H. (2019). Formation of Five-Membered Carbocycles from d -Glucose: A Concise Synthesis of 4-Hydroxy-2-(Hydroxymethyl)Cyclopentenone. Bull. Chem. Soc. Jpn..

[46] Grugel H., Albrecht F., Boysen M.M.K. (2014). *pseudo* Enantiomeric Carbohydrate Olefin Ligands—Case Study and Application in Kinetic Resolution in Rhodium(I)-Catalysed 1,4-Addition. Adv. Synth. Catal..

[47] Dixon J.T., van Heerden F.R., Holzapfel C.W. (2005). Preparation of an Analogue of Orbicuside A, an Unusual Cardiac Glycoside. Tetrahedron Asymmetry.

[48] Nitz M., Bundle D.R. (2001). Synthesis of Di- to Hexasaccharide 1,2-Linked β-Mannopyranan Oligomers, a Terminal S-Linked Tetrasaccharide Congener and the Corresponding BSA Glycoconjugates. J. Org. Chem..

[49] Peng H.-C., Bryan J., Henson W., Zhdankin V.V., Gandhi K., David S. (2019). New, Milder Hypervalent Iodine Oxidizing Agent: Using μ-Oxodi(Phenyliodanyl) Diacetate, a (Diacetoxyiodo)Benzene Derivative, in the Synthesis of Quinones. J. Chem. Educ..

[50] Qin C., Schumann B., Zou X., Pereira C.L., Tian G., Hu J., Seeberger P.H., Yin J. (2018). Total Synthesis of a Densely Functionalized *Plesiomonas shigelloides* Serotype 51 Aminoglycoside Trisaccharide Antigen. J. Am. Chem. Soc..

[51] Tokatly A.I., Vinnitsky D.Z., Kamneva A.A., Yashunsky D.V., Tsvetkov Y.E., Nifantiev N.E. (2023). Glycosylation with Derivatives of Phenyl 2-Azido-2-Deoxy-1-Seleno-α-d-Gluco- and -α-d-Mannopyranosides. Russ. Chem. Bull..

