# Supplementary material for: Optimization of Azidophenylselenylation of Glycals for the Efficient Synthesis of Phenyl 2-Azido-2-Deoxy-1-Selenoglycosides: Solvent Control
Source: Molecules. 2025 Dec 23;31(1):54. doi: 10.3390/molecules31010054 (PMC12786517; doi:10.3390/molecules31010054)
Supplement: Supplementary file 1 [file molecules-31-00054-s001.zip › molecules-3981325-supplementary.pdf]

Supplementary Information

Optimization of Azidophenylselenylation of Glycals for the Efficient Synthesis of 2-Azido-2-Deoxy-1-Selenoglycosides: Solvent Control

Bozhena S. Komarova [1], Olesia V. Belova [1], Timur M. Volkov [1], Dmitry V. Yashunsky [1] and Nikolay E. Nifantiev [1,*]

[1] *Laboratory of Glycoconjugate Chemistry, N.D. Zelinsky Institute of Organic Chemistry, Russian Academy of Sciences, Leninsky Prospect 47, 119991 Moscow, Russia.*

*Corresponding author: Nikolay E. Nifantiev. E-mail: nen@ioc.ac.ru

Table of Contents

1	**Experimental details**	S2
1.1	**Preparation of glycals 1 and 6 with guanidine-hydrochloride – Zn**	S2
1.2	**Preparation of L-fucal 20**	S3
1.3	**Preparation of L-rhamnal 25**	S4
1.4	**Preparation of glucal 30**	S5
1.5	**Synthesis of μ-*oxo*-BAIB from BAIB**	S7
1.6	**Investigation of Reaction Conversion at Different Temperatures**	S8
1.7	**Investigation into the influence of the solvent, substrate concentration (and the loading of all reagents), and the substrate:Ph_2Se_2 ratio on the outcome of the APS reaction of galactal and glucal**	S9
1.8	**Optimization of APS of 3-O-Benzyl-4,6-Benzylidene-D-Glucal 30**	S11
2	**NMR data**	S12
2.1	**NMR Data for compounds 1-9 and 16**	S12
2.2	**NMR Spectra of Crude Reaction Mixtures (Tables 1, S1, 2, 3)**	S14
3	**References**	S16

1 Experimental details

1.1 Preparation of glycals 1 and 6 with guanidine-hydrochloride – Zn

Conditions for the preparation of glycals **1** and **6** were newly optimized using guanidine hydrochloride as a base together with zinc, which in this case does not require any prior activation, in acetonitrile.

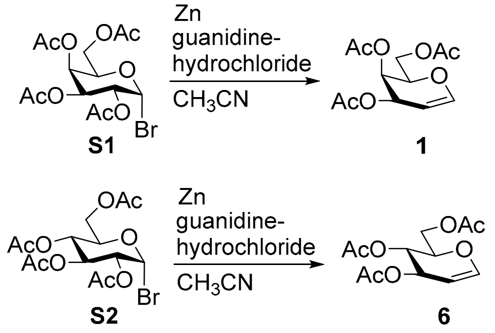

Scheme S1. Preparation of glycals **1** and **6**

Representative protocol for preparation of 3,4,6-tri-*O*-acetyl-D-galactal 1. Zn powder (6.1 g, 0.093 mol, without pre-activation) and guanidine hydrochloride (8.9 g, 98%, 0.091 mol) were successively added to a solution of acetobromo-α-D-galactose (5.09 g, 0.012 mol) in dry acetonitrile. The resulting reaction mixture was heated to 65 °C and stirred for 15 minutes. After completion of the reaction (TLC in toluene/acetone, 10:1), the mixture was diluted with EtOAc, filtered through a pad of Celite, and the filtrate was washed with brine. The aqueous phase was extracted with EtOAc three times, and the combined organic extracts were concentrated under reduced pressure. The resulting viscous oil was purified by silica gel chromatography on SiO_2 (petroleum ether-acetone gradient, 0→30%) to afford galactal **1** (2.40 g, 71%). Data for galactal (**1**): colorless oil; $R_f = 0.33$ (toluene-acetone, 10:1). ^1H NMR (600 MHz, $CDCl_3$): δ 6.45 (dd, $J_{1,2} = 6.3$ Hz, $J_{1,3} = 1.7$ Hz, 1H, H-1), 5.54 (m, 1H, H-3), 5.42 (m, 1H, H-4), 4.72 (m, 1H, H-2), 4.31 (m, 1H, H-5), 4.26 (dd, 1H, $J_{6A,5} = 7.4$ Hz, $J_{A,B} = 11.8$ Hz, H-6A), 4.21 (dd, 1H, $J_{6B,5} = 5.2$ Hz, $J_{A,B} = 11.8$ Hz, H-6B), 2.12, 2.08, 2.02 (3s, 3×1H, Ac). $^{13}C\{^1H\}$ NMR (150.9 MHz, $CDCl_3$): δ 170.5, 170.2, 170.1 (C=O (Ac)), 145.5 (C-1), 98.8 (C-2), 72.4 (C-5), 63.8 (C-3), 63.7 (C-4), 61.9

(C-6), 20.8, 20.7, 20.6 (CH_3(Ac)). The characterisation data are consistent with those reported for compound **1** in ref.[1]

3,4,6-tri-*O*-acetyl-D-glucal 6 was prepared using the same protocol. Data for glucal (**6**): colorless oil; R_f = 0.20 (toluene/EtOAc, 10:1). ^{1}H NMR (300 MHz, $CDCl_3$): δ 6.47 (dd, $J_{1,2}$ = 6.0 Hz, $J_{1,3}$ = 1.2 Hz, 1H, H-1), 5.34 (m, 1H, H-3), 5.23 (dd, J = 5.6, 7.4 Hz, 1H, H-4), 4.85 (dd, J = 3.2, 6.2 Hz, 1H, H-2), 4.41 (dd, $J_{6A,6B}$ = 11.8 Hz, $J_{6A,5}$ = 5.4 Hz, H-6A), 4.30–4.17 (m, 2H, H-5, H-6B), 2.10, 2.08, 2.05 (3s, 3×1H, Ac). ^{13}C{^{1}H} NMR (75.5 MHz, $CDCl_3$): δ 170.7, 170.6, 169.7 (C=O (Ac)), 145.8 (C-1), 99.1 (C-2), 67.6 (C-3), 67.3 (C-4), 61.5 (C-6), 21.1, 20.90, 20.85 (CH_3(Ac)). The characterisation data are consistent with those reported for compound **6** in ref [2].

1.2 Preparation of L-fucal 20

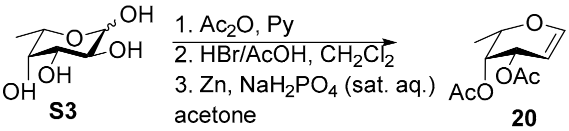

Scheme S2. Preparation of L-fucal **20**

3,4-di-*O*-acetyl-L-fucal 20. To a solution of L-fucopyranoside **S3** (10.52 g, 64.15 mmol) in pyridine (80.00 mL) acetic anhydride (40.00 mL, 423.53 mmol) was added. The mixture was left overnight and coevaporated with toluene several times. The dry residue was dissolved in CH_2Cl_2 (170.00 mL), the solution was chilled to 0 °C and 33% HBr in AcOH (33.70 mL) was added dropwise and the mixture was allowed to warm up to room temperature. After 1 hour the mixture was washed with ice water, the aqueous phase was washed twice with CH_2Cl_2, combined organic phase was washed with saturated aqueous $NaHCO_3$. Then the solvent was evaporated and the dry residue was dissolved in acetone (139.00 mL), then 4.6M aqueous NaH_2PO_4 (209.00 mL) and Zn (29.12 g, 448 mmol) were added while vigorous stirring. After 40 min the mixture was filtered through celite pad, the filter was washed with CH_2Cl_2 and filtrate was washed with water. Aqueous phase was washed twice with CH_2Cl_2, combined organic phase was concentrated *in vacuo*. Column chromatography on SiO_2 (petroleum ether-EtOAc gradient, 5→10%) afforded

fucal **20** as a colorless solid (8.86 g, 65%). ^{1}H NMR (300 MHz, $CDCl_3$): δ 6.45 (dd, $J_{1,2}$ = 6.3, $J_{1,3}$ = 1.8 Hz, 1H, H-1), 5.59 – 5.53 (m, 1H, H-3), 5.30 – 5.25 (m, 1H, H-4), 4.63 (dt, $J_{2,1}$ = 6.3, $J_{2,3}$ = 1.9 Hz, 1H, H-2), 4.20 (q, $J_{5,6}$ = 6.6 Hz, 1H, H-5), 2.14 (s, 3H, Ac), 2.00 (s, 3H, Ac), 1.26 (d, $J_{6,5}$ = 6.6 Hz, 3H, H-6). ^{13}C{^{1}H} NMR (75.5 MHz, $CDCl_3$): δ 170.7, 170.4, 146.1 (C-1), 98.2 (C-2), 71.5 (C-5), 66.2 (C-4), 65.0 (C-3), 20.8 (CH_3C(O)), 20.7 (CH_3C(O)), 16.5 (C-6). The characterisation data are consistent with those reported for compound in ref [3].

1.3 Preparation of L-rhamnal 25

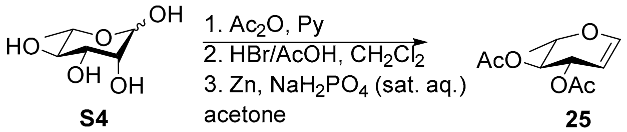

Scheme S3. Preparation of L-rhamnal **25**

3,4-di-*O*-acetyl-L-rhamnal 25. To a solution of L-rhamnopyranoside **S4** (4.00 g, 24.39 mmol) in pyridine (30.00 mL) acetic anhydride (13.80 mL, 146.12 mmol) was added. The mixture was left overnight and coevaporated with toluene several times. The dry residue was dissolved in CH_2Cl_2 (70.00 mL), the solution was chilled to 0 °C and 33% HBr in AcOH (12.80 mL) was added dropwise and the mixture was allowed to warm up to room temperature. After 1 hour the mixture was washed with ice water, the aqueous phase was washed twice with CH_2Cl_2, combined organic phase was washed with saturated aqueous $NaHCO_3$. Then the solvent was evaporated and the dry residue was dissolved in acetone (50 mL), then 4.6M aqueous NaH_2PO_4 (81.62 mL) and Zn (11.12 g, 171 mmol) were added while vigorous stirring. After 1 h 20 min the mixture was filtered through schott filter, the filter was washed with EtOAc, filtrate was washed with brine and dried upon Na_2SO_4. Organic phase was concentrated *in vacuo*. Column chromatography on SiO_2 (petroleum ether-EtOAc gradient, 0→10%) afforded rhamnal **25** as a colorless liquid (3.70 g, 71%). ^{1}H NMR (300 MHz, $CDCl_3$): δ 6.43 (dd, $J_{1,2}$ = 6.1, $J_{1,3}$ = 1.2 Hz, 1H, H-1), 5.34 (ddd, $J_{3,4}$ = 6.1, $J_{3,2}$ = 3.0, $J_{3,1}$ = 1.2 Hz, 1H, H-3), 5.06 – 5.00 (m, 1H, H-4), 4.78 (dd, $J_{2,1}$ = 6.1, $J_{2,3}$ = 3.0 Hz, 1H, H-2), 4.16 – 4.06 (m, 1H, H-5), 2.09 (s, 3H, Ac), 2.05 (s, 3H, Ac), 1.31 (d, $J_{6,5}$ = 6.5 Hz, 3H, H-6). ^{13}C{^{1}H} NMR (75.5 MHz, $CDCl_3$): δ 170.6, 169.8, 145.9 (C-1), 98.7 (C-2), 72.5

(C-5), 71.8 (C-4), 68.2 (C-3), 21.0 ($CH_3C(O)$), 20.9 ($CH_3C(O)$), 16.5 (C-6). The characterisation data are consistent with those reported for compound in ref [4].

1.4 Preparation of glucal 30

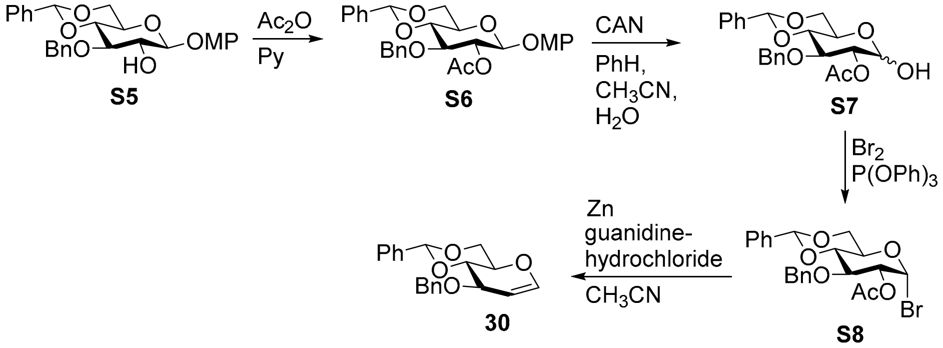

Scheme S4. Preparation of glucal **30**

***p*-Methoxyphenyl 2-*O*-acetyl-3-*O*-benzyl-4,6-*O*-benzyliden-*β*-D-glucopyranoside (S6).** Under argon atmosphere to a solution of *p*-methoxyphenyl 3-*O*-benzyl-4,6-*O*-benzyliden-*β*-D-glucopyranoside (13.6 g, 0.029 mol) in dry CH_2Cl_2 (200 mL) were added acetic anhydride (19.2 mL, 0.204 mol) and pyridine (54 mL, 0.672 mol). After 48 h the mixture was concentrated *in vacuo*, the residue was dissolved in EtOAc and washed with 0.1M H_2SO_4 and saturated aqueous $NaHCO_3$, the organic layer was dried upon Na_2SO_4 and concentrated. Crystallization of dry residue from ethanol afforded white crystals of **S6** (11.4 g), crystallization of concentrated mother liquor afforded additionally 0.6 g of **S6**, thus the total yield was 81%. Data for glucopyranoside (**S6**): white crystals; mp +144-146°C (EtOH); optical rotation $[\alpha]_D$ +10.95 (c 1.00 in $CHCl_3$). ^1H NMR (300 MHz, $CDCl_3$): δ = 7.57-7.51(m, 2H, Ph), 7.48-7.39 (m, 3H, Ph), 7.37-7.28 (m, 5H, Ph), 6.99-6.96 (m, 2H. Ph(MP)), 6.88-6.82 (m, 2H, Ph(MP), 5.64 (s, 1H, PhC*H*), 5.33-5.28 (m, 1H, H-2), 4.99-4.93 (m, 2H, H-1, BnA), 4.75 (d, 1H, J_{AB} = 12.09 Hz, BnB), 4.43 (dd, 1H, $J_{6A,6B}$ = 10.5 Hz, $J_{6A,5}$ = 4.9 Hz, H-6A), 3.94-3.83 (m, H-3, H-4, H-6B), 3.8 (s, 3H, OMe), 3.62-3.53 (m, 1H, H-5). $^{13}C\{^1H\}$ NMR (75.5 MHz, $CDCl_3$): δ = 169.3, 155.7, 151.1, 138.2, 137.2, 129.1, 128.4, 128.3, 127.9, 127.7, 126.1, 118.6, 114.6, 101.4 (Ph*C*H), 101.1 (C-1), 81.4 (C-4), 78.4 (C-3), 77.5, 77.1, 76.7, 74.2, 72.8 (C-2), 68.7 (C-6), 66.4 (C-5), 55.7 (OMe), 20.9 (Ac). HRMS ESI m/z calcd for [M+Na]+ $C_{29}H_{30}NaO_8^+$ 529.1833; found 529.1833.

2-*O*-acetyl-3-*O*-benzyl-4,6-*O*-benzyliden-*β*-D-glucopyranose (S7). *p*-Methoxyphenyl 2-*O*-acetyl-3-*O*-benzyl-4,6-*O*-benzyliden-*β*-D-glucopyranoside (**S6**) (3.00 g, 5.93 mmol) was dissolved in a mixture of MeCN (210 mL) and benzene (30 mL), the resulting solution was cooled to –10 °C and CAN (16.25 g, 29.65 mmol) in water (60 mL) was added. After 7 minutes reaction mixture was poured into saturated aqueous $NaHCO_3$ and washed three times with EtOAc, combined organic layers were dried upon Na_2SO_4 and concentrated under reduced pressure. Column chromatography of residue on SiO_2 (toluene-EtOAc gradient, 0→33%) afforded **S7** (1.95 g, 82%) as colorless crystals. Data for glucopyranose (**S7**): colorless crystals. ^{1}H NMR (300 MHz, $CDCl_3$): δ 7.52-7.44 (m, 3H), 7.43-7.21 (m, 12H), 5.60-5.55 (m, 1.4H, PhC*H*), 5.40 (d, 1H, $J_{1\alpha,2\alpha}$ = 3.8 Hz, H-1$^\alpha$), 4.93-4.80 (m, 2.8H, BnA$^\alpha$, BnA$^\beta$, H-2$^\alpha$, H-2$^\beta$), 4.74-4.62 (m,1.8H, BnB$^\alpha$, BnB$^\beta$, H-1$^\beta$), 4.36 (dd, 0.4H, $J_{6A\beta,\,6B\beta}$ = 10.5 Hz, $J_{6A\beta,\,5\beta}$ = 5.0 Hz, H-6A$^\beta$), 4.28 (dd, 1H, $J_{6A\alpha,\,6B\alpha}$ = 10.2 Hz, $J_{6A\alpha,\,5\alpha}$ = 5 Hz, H-6A$^\alpha$), 4.15-4.03 (m, 2H, H-5$^\alpha$, H-3$^\alpha$), 3.80-3.67 (m, 3.2H, H-6B$^\beta$, H-3$^\beta$, H-4$^\beta$, H-4$^\alpha$, H-6B$^\alpha$), 3.51-3.41 (m, 0.4H, H-5$^\beta$), 2.09-2.04 (m, 4.2H, Ac$^\alpha$, Ac$^\beta$). ^{13}C{^{1}H} NMR (75.5 MHz, $CDCl_3$): δ 171.5, 170.5, 138.4, 137.3, 137.1, 129.1, 129.0, 128.4, 128.3, 127.9, 127.8, 127.7, 126.1, 126.0, 101.4 (Ph*C*H$^\alpha$), 101.3 (Ph*C*H$^\beta$), 96.3 (C-1$^\beta$), 91.1 (C-1$^\alpha$), 82.1 (C-3$^\alpha$), 81.6 (C-3$^\beta$), 78.0 (C-4$^\beta$), 77.5, 77.1, 76.7, 75.8 (C-5$^\alpha$), 75.6 (C-2$^\beta$), 74.9 (Bn$^\alpha$), 74.5 (Bn$^\beta$), 73.2 (C-2$^\alpha$), 69.0 (C-6$^\alpha$), 68.6 (C-6$^\beta$), 66.5 (C-5$^\beta$), 62.5 (C-4$^\alpha$), 20.9 (Ac). HRMS ESI m/z calcd for $[M+Na]^+$ $C_{22}H_{24}NaO_7^+$ 423.1414, found 423.1406. The characterisation data are consistent with those reported for compound in ref [5].

2-*O*-acetyl-3-*O*-benzyl-4,6-*O*-benzyliden-*β*-D-glucopyranoside bromide (S8). Under argon atmosphere at –20 °C bromine (352 µL, 7.00 mmol) was dissolved in dry CH_2Cl_2 (4.00 mL), a solution of $(PhO)_3P$ (1.84 mL, 7.00 mmol) in dry CH_2Cl_2 (2.00 mL) was added. The resulting mixture was allowed to warm up to room temperature within 30 min. The resulting colorless mixture was added to a solution of 2-*O*-acetyl-3-*O*-benzyl-4,6-*O*-benzyliden-*β*-D-glucopyranose (**S7**) (2.00 g, 5.00 mmol) in dry CH_2Cl_2 (8.00 mL) and pyridine (522 µL, 6.5 mmol). After 10 min the reaction was quenched with saturated aqueous $NaHCO_3$, diluted and washed three times

with CH_2Cl_2, combined organic layers were concentrated *in vacuo*, column chromatography of the residue on SiO_2 (toluene-ethylacetate gradient, 0→2%) afforded bromide **S8** as a colorless syrup. Data for bromide (**S8**): ^{1}H NMR (300 MHz, $CDCl_3$) δ 7.54 – 7.46 (m, 2H), 7.43 – 7.10 (m, 36H), 6.88 – 6.80 (m, 0.6H), 6.77 – 6.70 (m, 1H), 6.62 (d, $J_{1,2}$ = 4.1 Hz, 1H, H-1), 5.59 (s, 1H, PhC*H*), 4.90 (d, *J* = 11.7 Hz, 1H, BnA), 4.83 – 4.70 (m, 2H, H-2, BnB), 4.33 (dd, $J_{6A, 6B}$ = 10.2, $J_{6A, 5}$ = 4.9 Hz, 1H, H-6A), 4.23 – 4.06 (m, 2H, H-5, H-3), 3.86 – 3.75 (m, 2H, H-4, H-6B), 2.08 (s, 1H, Ac). ^{13}C{^{1}H} NMR (75.5 MHz, $CDCl_3$) δ 170.0, 150.4, 150.3, 138.0, 136.8, 129.8, 129.3, 129.1, 128.3, 128.2, 127.7 (×2), 126.0, 125.6, 120.1, 120.0, 119.9, 115.3, 101.5 (Ph*C*H), 88.3 (C-1), 80.7 (C-4), 76.0 (C-3), 75.0 (Bn), 72.9 (C-2), 67.9 (C-6), 67.0 (C-5), 20.7 (*C*H_3C(O)).

3-*O*-benzyl-4,6-*O*-benzyliden-D-glucal (30)

Under argon atmosphere bromide **S8** was dissolved in dry MeCN (50 mL), Zn (2.9 g, 0.045 mol) and guanidine hydrochloride (3.6 g, 0.038 mol) were added. The resulting reaction mixture was heated to 80 °C and stirred for 35 minutes. After completion of the reaction (TLC in petroleum ether/ethylacetate, 10:1), the mixture was diluted with EtOAc, filtered through a pad of Celite, and the filtrate was washed with brine. The aqueous phase was extracted with EtOAc three times, and the combined organic extracts were concentrated under reduced pressure. Column chromatography on SiO_2 (petroleum ether-EtOAc gradient, 0→5%) of residue afforded glucal **30** (859 mg, 53%). Data for glucal (**30**): white crystals; mp +107-109°C; optical rotation $[\alpha]_D$ –41.3 (c 1.00 in $CHCl_3$); ^{1}H NMR (300 MHz, $CDCl_3$): δ 7.54-7.23 (m, 10H, Ph, Bn), 6.35 (dd, 1H, *J* = 6.2 Hz, *J* = 1.6 Hz, H-1), 5.63 (s, 1H, PhCH), 4.85-4.78 (m, 2H, H-2, BnA), 4.71 (d, 1H, *J* = 12.12 Hz, BnB), 4.40-4.33 (m, 2H, H-3, H-6A), 4.07-3.99 (m, 1H, H-4), 3.96-3.79 (m, 2H, H-5, H-6B) .^{13}C{^{1}H} NMR (75.5 MHz, $CDCl_3$): δ 144.4 (C-1), 138.5, 137.3, 129.0, 128.4, 128.3, 127.8, 127.6, 126.1, 102.3 (C-2), 101.2 (Ph*C*H), 80.0 (C-4), 77.5, 77.0, 76.6, 73.1 (C-3), 72.0 (Bn), 68.7 (C-5), 68.4 (C-6). HRMS ESI m/z calcd for $[M+NH_4]^+$ $C_{20}H_{24}NO_4^+$ 342.1700; found 342.1698.

1.5 Synthesis of μ-*oxo*-BAIB from BAIB

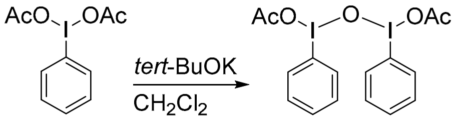

Scheme S5. Synthesis of μ-*oxo*-BAIB

μ-*Oxo*-bis(acetyloxy)iodobenzene. Prior to the reaction, BAIB was dissolved in CH_2Cl_2 (11 g/mL), and the resulting turbid solution was filtered through Celite and concentrated under reduced pressure. The residue was then dissolved in CH_2Cl_2, and hexane (7:3) was added. The precipitated crystals were collected by filtration and used for BAIB preparation. Under an argon atmosphere, recrystallized BAIB (980 mg) was added to a suspension of *tert*-BuOK (374 mg) in dry CH_2Cl_2 (20 mL). After 4 h, the reaction mixture was filtered through a triple-layered filter consisting of Celite between layers of glass wool. The resulting solution was concentrated, and the residue was dissolved in CH_2Cl_2 (0.8 mL), diluted with hexane (4 mL), and the solvent decanted. This procedure was repeated three times. The remaining solid was dried to afford μ-*oxo*-BAIB (470 mg, ~56%) containing approximately 20% BAIB. Data for μ-*oxo*-BAIB: white crystals, ^1H NMR (600 MHz, $CDCl_3$): δ 7.84 (d, J = 8.0 Hz, 2H, Ph), 7.48 (t, J = 7.3 Hz, 1H, Ph), 3.56 (t, J = 7.8 Hz, 2H, Ph), 1.90 (s, 3H, Ac).

1.6 Investigation of Reaction Conversion at Different Temperatures

Under an argon atmosphere at −5 °C, a solution of galactal (332.0 mg, 1.22 mmol) in dry propionitrile (17 mL) was successively treated with NaN_3 (161.2 mg, 2.48 mmol), BAIB (472.3 mg, 1.47 mmol), and Ph_2Se_2 (571.4 mg, 1.83 mmol). The reaction mixture was then allowed to warm to 25 °C over 40 minutes and was stirred further. After two days, as indicated by TLC (toluene/EtOAc, 7:1), the reaction ceased with incomplete conversion, and the accumulation of side products was observed. The reaction mixture was diluted with EtOAc and washed with a 1:1 mixture of saturated aqueous of $NaHCO_3$ and 10% $Na_2S_2O_3$. The aqueous layer was subsequently extracted three times with additional EtOAc, and the combined organic extracts were concentrated under reduced pressure. The dry residue was purified by column

chromatography on SiO_2 (toluene-acetone gradient, 0→3%), yielding a sample of phenylseleno 2-azidogalactoside (417.1 mg, <73%). NMR analysis revealed that the sample contained 8% of the talo isomer, 8% of azido 2-phenylseleno-2-deoxy-taloside, and 7% of azido 2-phenylseleno-2-deoxy-galactoside.

A reaction at 15 °C was carried out in a similar manner: at −5 °C, a solution of galactal (1.12 g, 4.11 mmol) in propionitrile (54 mL) was successively treated with NaN_3 (534.5 mg, 8.2 mmol), BAIB (1.59 g, 4.94 mmol), and Ph_2Se_2 (1.92 g, 6.15 mmol). The resulting mixture was then stirred at 15 °C in a thermostatted bath. After one day, the reaction was complete. The mixture was worked up and purified by column chromatography, affording 1.68 g (<87%) of phenylseleno 2-azidogalactoside, which contained the same amount of side-product impurities as previously observed.

1.7 Investigation into the influence of the solvent, substrate concentration (and the loading of all reagents), and the substrate:Ph_2Se_2 ratio on the outcome of the APS reaction of galactal and glucal.

To standardize the reaction conditions, seven reactions were performed in parallel in a thermostatically controlled bath, using identical tightly sealed test tubes equipped with identical magnetic stir bars. In all cases, the solvent volume was 2 mL. The glycal concentration in the reactions was either 0.21 mmol/mL or 0.07 mmol/mL. To achieve this, stock solutions of glycal (0.21 mmol/mL) were prepared and then diluted as needed. Dry solvents were used, and the test tubes containing the reaction mixture were purged with argon.

To the glycal solution in the test tube cooled to –15 °C, NaN_3 (0.29 or 0.84 mmol) was first added, followed by BAIB (0.15 or 0.51 mmol). The amounts of reagents are provided in Table S2 in Supporting information. After 20–30 minutes of stirring, Ph_2Se_2 (0.09 or 0.25 mmol) was introduced into the reaction mixture. The progress of the reaction was monitored by TLC (toluene/EtOAc, 7:1). Once the reaction was complete, or after it was determined that no reaction occurred over 24 hours, the contents of the test tube were diluted 4–5 times with EtOAc and

transferred to a separatory funnel containing an aqueous mixture of 10% $Na_2S_2O_3$ and saturated $NaHCO_3$ (1:1). The funnel was shaken, and after separation of the organic phase, the aqueous phase was washed three additional times with EtOAc. The combined organic extracts were concentrated under reduced pressure, and the dry residue was analyzed by NMR. In cases where TLC indicated a yield above 75%, the products were purified by column chromatography on SiO_2 (toluene-acetone gradient, 0→3% for the galactose derivative; toluene-EtOAc gradient, 0→20% for the glucose derivative).

Table S1. Selected APS of galactal **1** (in addition to Table 1, Scheme 1) and outcome determined by NMR

Entry	Solvent[a]	[Glycal]/ mmol·mL^{-1}	t/d	Conv./%	Yield/%	Gal:Tal	2-N_3:2-SePh
S1	CH_2Cl_2	0.07	4	90	74	10.4:1	10:1
S2	PhH[b]	0.07	1.5	100	80	7.8:1	8.8:1
S3	CH_3CN	0.07	5	100	80	8.2:1	8.8:1
S4	PhH-pentane (1:1)	0.21	4	95	74	11.8:1	5.5:1
S5	PhH-hexane (1:1)	0.21	4	95	74	10.6:1	5.9:1

[a]All reactions were carried out at 15 °C under argon, with 0.6 equiv Ph_2Se_2 and 2.0 equiv BAIB. [b]PhH - benzene

Table S2. Reagent quantities, solvent system, and substrate concentration for azidophenylselenylations of galactal **1** presented in Tables 1–2

Entry	Solvent[a]	Conc. (mmol/2mL)	NaN_3 (mg, mmol)	BAIB (mg, mmol)	Ph_2Se_2 (mg, mmol)
Table 1, 1	CH_2Cl_2	0.42	54.0 (0.83)	165.7 (0.51)	79.3 (0.25)
Table 1, 2	PhH	0.42	54.8 (0.84)	163.6 (0.51)	80.1 (0.26)
Table 1, 3	CH_3CN	0.42	54.6 (0.84)	162.3 (0.50)	78.7 (0.25)
Table 1, 4	EtCN	0.42	56.1 (0.86)	162.9 (0.51)	80.4 (0.26)
Table 1, 5	PhH/heptane (1:1)	0.42	55.5 (0.85)	163.8 (0.51)	79.1 (0.25)
Table 1, 6	PhH/EtCN/heptane (1:1:1)	0.42	54.6 (0.84)	162.3 (0.50)	78.7 (0.25)
Table 2, 1	PhH (TMSOTf, 6.3 µL, 4% mol to NaN_3)	0.42	55.8 (0.86)	162.9 (0.51)	79.8 (0.26)
Table 2, 2	PhH (TMSCl, 4 µL, 4% mol to NaN_3)	0.42	59.6 (0.92)	164.8 (0.51)	79.3 (0.25)
Table 2, 4	PhH/EtCN/heptane (1:1:1) (TMSCl, 4 µL, 4% mol to NaN_3)	0.42	55.6 (0.86)	163.6 (0.51)	78.9 (0.25)

[a]PhH - benzene

Table S3. Reagent quantities, solvent system, and substrate concentration for azidophenylselenylations of glucal **6** presented in Table 3

Entry	Solvent[a]	Conc. (mmol/2mL)	NaN_3 (mg, mmol)	BAIB (mg, mmol)	Ph_2Se_2 (mg, mmol)
Entry 1	PhH	0.42	54.6 (0.84)	162.3 (0.50)	78.7 (0.25)
Entry 2	PhH	0.14	19.2 (0.29)	49.7 (0.15)	27.3 (0.09)

Entry 3	PhH/heptane (1:1)	0.42	55.2 (0.85)	164.0 (0.51)	79.0 (0.25)
Entry 4	CH_3CN	0.42	55.9 (0.86)	162.7 (0.51)	79.2 (0.25)

[a]PhH - benzene

Table S4. Reagent quantities, solvent system, and substrate concentration for azidophenylselenylations of galactal **1** presented in Table S1

**Entry**	**Solvent**[a]	**Conc. (mmol/2mL)**	**NaN_3 (mg, mmol)**	**BAIB (mg, mmol)**	**Ph_2Se_2 (mg, mmol)**
Entry S1	CH_2Cl_2	0.14	21.1 (0.32)	55.7 (0.17)	28.1 (0.09)
Entry S2	PhH	0.14	19.1 (0.29)	55.1 (0.17)	28.4 (0.09)
Entry S3	CH_3CN	0.14	21.3 (0.32)	55.7 (0.17)	27.8 (0.09)
Entry S4	PhH/pentane (1:1)	0.42	54.6 (0.84)	162.3 (0.50)	78.7 (0.25)
Entry S5	PhH/hexane (1:1)	0.42	56.1 (0.86)	162.8 (0.51)	79.5 (0.25)

1.8 Optimization of APS of 3-O-Benzyl-4,6-Benzylidene-D-Glucal 30

Table 4, Entry 1

Ph_2Se_2 (102 mg, 0.33 mmol) and NaN_3 (30 mg, 0.46 mmol) were added to a solution of glucal **30** (70 mg, 0.22 mmol) in dry EtCN (5 mL) under argon while stirring. Then the mixture was cooled to −10 °C and BAIB (83 mg, 0.26 mmol) was added. Reaction mixture was allowed to warm up to room temperature, then TMSCl (9 μL, 0.07 mmol) was added. After 3 days starting material was not fully consumed, the mixture was diluted by CH_2Cl_2 and washed with aqueous saturated $NaHCO_3$. The aqueous layer was washed twice with CH_2Cl_2, combined organic phase was concentrated *in vacuo* and residue was purified by column chromatography (petroleum ether-EtOAc gradient, 40:1→15:1) to produce phenyl 2-azido-3-*O*-benzyl-4,6-*O*-benzylidene-2-deoxy-1-seleno-α-glucopyranoside **31** (5 mg, 4%).

Table 4, Entry 2

Ph_2Se_2 (50 mg, 0.16 mmol) and NaN_3 (17 mg, 0.27 mmol) were added to a solution of glucal **30** (43 mg, 0.13 mmol) in dry EtCN (3 mL) under argon while stirring. Then the mixture was cooled to -20°C and mixture of BAIB and μ-oxo-BAIB (1:1) (69 mg) was added. Reaction mixture was allowed to warm up to 10°C. After stirring for 24 h at this temperature the mixture was diluted by CH_2Cl_2 and washed with aqueous saturated $NaHCO_3$. The aqueous layer was washed twice with CH_2Cl_2, combined organic phase was concentrated *in vacuo* and residue was

purified by column chromatography (petroleum ether-EtOAc gradient, 40:1→15:1) to produce phenyl 2-azido-3-*O*-benzyl-4,6-*O*-benzylidene-2-deoxy-1-seleno-α-D-glucopyranoside **31** (15 mg, 22%).

Table 4, Entry 3

The solution of glucal **30** (40 mg, 0.12 mmol) and Ph_2Se_2 (38 mg, 0.12 mmol) in dry CH_2Cl_2 (1.4 mL) under argon was cooled to -30°C. $TMSN_3$ (32 μl, 0.25 mmol) and BAIB (40 mg, 0.12 mmol) were added sequentially while stirring. When the reaction mixture became homogeneous it was placed in freezer at -25°C for 24h. Then the mixture was diluted by CH_2Cl_2 and washed with aqueous saturated $NaHCO_3$. The aqueous layer was washed twice with CH_2Cl_2, combined organic phase was concentrated *in vacuo* and residue was purified by column chromatography (petroleum ether-EtOAc gradient, 40:1→15:1) to produce colorless crystals of phenyl 2-azido-3-*O*-benzyl-4,6-*O*-benzylidene-2-deoxy-1-seleno-α-D-glucopyranoside **31** (42 mg, 65%). Data for 2-azido-3-*O*-benzyl-4,6-*O*-benzylidene-2-deoxy-1-seleno-α-D-glucopyranoside (**31**): mp +119 – 121°C ; optical rotation $[\alpha]_D$ + 142.76 (c 1.00 in $CHCl_3$). ^{1}H NMR (500 MHz, $CDCl_3$) δ 7.66 – 7.62 (m, 2H), 7.57 – 7.52 (m, 2H), 7.47 – 7.30 (m, 11H), 5.90 (d, *J* = 4.6 Hz, 1H, H-1), 5.63 (s, 1H, PhC*H*), 5.02 (d, *J* = 10.9 Hz, 1H, BnA), 4.87 (d, *J* = 10.9 Hz, 1H, BnB), 4.36 (td, *J* = 9.9, 5.0 Hz, 1H, H-5), 4.18 (dd, *J* = 10.4, 5.0 Hz, 1H, H-6A), 4.00 – 3.94 (m, 2H, H-3, H-2), 3.85 – 3.76 (m, 2H, H-4, H-6B). ^{13}C{^{1}H} NMR (126 MHz, $CDCl_3$) δ 135.0, 129.2, 129.1, 128.5, 128.3, 128.2, 128.0, 126.0, 101.5 (Ph*C*H), 84.9 (C-1), 82.3 (C-4), 78.8 (C-3), 75.2 (Ph*C*H_2), 68.4 (C-6), 65.3 (C-5), 64.2 (C-2). HRMS ESI m/z calcd for [M+H]+ $C_{26}H_{26}N_3O_4Se^+$ 524.1085; found 524.1079; The characterisation data are consistent with those reported for this compound in ref [6].

2 NMR data

2.1 NMR Data for compounds 1-9 and 16

Data for phenyl 3,4,6-tri-O-acetyl-2-azido-2-deoxy-1-seleno-α-D-galactoside (**2**) and byproducts **3**, **4**, and **5**: ^{1}H NMR (600 MHz, $CDCl_3$): δ 7.62–7.52 (m, 2.2H, PhSe), 7.34–7.24 (m, 3.86H, PhSe), 6.00 (d, $J_{1,2}$ = 5.5 Hz, 1H, H-1^{2}), 5.87 (br s, 0.079H, H-1^{3}), 5.70 (br s, 0.050H, H-1^{5}), 5.65 (d, $J_{1,2}$ = 4.0 Hz, 0.083H, H-1^{4}), 5.46 (d, $J_{5,6}$ = 3.2 Hz, 1H, H-4^{2}), 5.11 (dd, $J_{3,4}$ = 3.3 Hz, $J_{3,2}$ = 10.8 Hz, 1H, H-3^{2}), 5.44–5.25 (m, 0.53H, H-4^{3}, H-4^{4}, H-3^{5}), 4.73–4.66 (m, H-5^{3}), 4.66 (t, $J_{5,6}$ = 6.5 Hz, 1H, H-5^{2}), 4.39 (br t, 0.13H, H-5^{4,5}), 4.25 (dd, $J_{2,1}$ = 5.5 Hz, $J_{2,3}$ = 10.8 Hz, 1H. H-2^{2}), 4.22–3.98 (m, 2.62H, H-6^{2,3,4,5}), 3.56 (dd, $J_{2,1}$ = 4.4Hz, $J_{2,3}$ = 11.9 Hz, 0.078H, H-2^{4}), 3.42 (br d, $J_{2,3}$ = 5.1 Hz, 0.052H, H-2^{5}), 2.21, 2.18, 2.14, 2.08, 2.05, 2.04, 2.00, 1.97, 1.88 (9×s, 11.0H, CH_3(Ac)). ^{13}C{^{1}H} NMR (150.9 MHz, $CDCl_3$): 170.0, 169.7 (C=O(Ac)), 134.9, 129.3, 128.3 (Ph), 91.7 (C-1^{3}), 91.0 (C-1^{4}), 84.2 (C-1^{2}), 83.0 (C-1^{5}), 71.3 (C-3^{2}), 69.8 (C-5^{3}, C-3^{4}), 69.3 (C-5^{4,5}), 69.1 (C-5^{2}), 68.4 (C-3^{3}), 67.7 (C-4^{4}), 66.8 (C-3^{5}), 66.3 (C-4^{5}), 65.9 (C-4^{3}), 61.9, 61.6 (C-6^{2,3,4,5}), 59.8 (C-2^{3}), 58.8 (C-2^{2}), 46.0 (C-2^{5}), 43.5 (C-2^{4}), 20.7 (Ac). The spectrum exhibits signals attributable to compounds **2**, **3**, **4**, and **5** that are consistent with those previously reported in ref.[7]

Data for 1,3,4,6-tetra-O-acetyl-2-deoxy-2-phenylseleno-D-galactosides and 1,3,4,6-tetra-O-acetyl-2-deoxy-2-phenylseleno-*α*-D-taloside (**16**): ^{1}H NMR (600 MHz, $CDCl_3$): δ 7.70–7.67 (d, *J* = 7.5 Hz, 4.4H, Ar), 7.60–7.51 (m, 5.0 Hz, Ar), 7.36–7.21 (m, 10.4H, Ar), 7.09 (t, *J* = 7.8 Hz, 4.5Hz, Ar), 6.47 (br s, 0.17H, H-1^{Tal}), 6.44 (d, $J_{1,2}$ = 3.5 Hz, 1H, H$^{\alpha}$), 5.70 (d, $J_{1,2}$ = 9.5 Hz, 0.95H, H-1$^{\beta}$), 5.43–5.36 (m, 2.64H, H-4^{Tal}, H-3^{Tal}, H-4$^{\alpha}$, H-3$^{\alpha}$), 5.28 (d, $J_{4,3}$ = 3.2 Hz, 0.95H, H-4$^{\beta}$), 4.79 (dd, $J_{3,4}$ = 3.2 Hz, $J_{3,2}$ = 12.0 Hz, 0.95H, H-3$^{\beta}$), 4.35 (br t, $J_{5,6}$ = 6.8 Hz, 0.20H, H-5^{Tal}), 4.13–4.01 (m, 6.37 H, H-6$^{\alpha\beta}$), 3.58 (dd, $J_{2,1}$ = 3.5 Hz, $J_{2,3}$ = 12.0 Hz, 1H, H-2$^{\alpha}$), 3.48 (br d, $J_{2,3}$ = 5.2 Hz, H-2), 3.40 (dd, $J_{2,3}$ = 12.0 Hz, $J_{2,1}$ = 9.5 Hz, 0.95H, H-2$^{\beta}$), 2.14–1.94 (9×s, Ac). ^{13}C{^{1}H} NMR (150.9 MHz, $CDCl_3$): δ 170.3, 170.0, 169.8, 169.5, 168.7 (C=O (Ac)), 137.4, 136.3, 134.5, 130.2, 129.2, 128.9, 128.1, 127.4 (Ar), 95.8 (C-1^{Tal}), 93.6 (C-1$^{\beta}$), 92.9 (C-1$^{\alpha}$), 71.4 (C-5$^{\beta}$), 69.9 (C-3$^{\beta}$), 69.3 (C-3$^{\alpha}$), 68.5 (C-5$^{\alpha}$), 67.2 (C-4$^{\alpha}$), 66.3 (C-4$^{\beta}$), 61.3, 61.1 (C-6), 45.1 (C-2^{Tal}), 43.0 (C-2$^{\alpha}$), 42.5 (C-2$^{\beta}$), 20.7, 20.6, 20.5 (CH_3 (Ac)).

Table S5. ^{1}H-NMR data for compounds **1-9**, **16** ($CDCl_3$)

**Compound**	**H1 ($J_{1,2}$)**	**H2 ($J_{2,3}$)**	**H3 ($J_{3,4}$)**	**H4 ($J_{4,5}$)**	**H5 ($J_{5,6A}$)**	**H6A ($J_{6A,6B}$)**	**H6B ($J_{6B,5}$)**
**1**	6.45 (6.3) ($J_{1,3}$ 1.7)	4.72	5.54	5.42	4.31 (7.4)	4.26 (11.8)	4.21 (5.2)
**2**	5.99 (5.4)	4.25 (10.9)	5.11 (3.2)	5.46 (3.3)	4.66 (6.5)	4.04	4.04
**3***	5.87	4.05	5.30	5.42	4.69	4.17	4.17
**4***	5.65 (4.1)	3.56 (11.7)	5.22 (3.2)	5.36	4.39 (6.6)		
**5***	5.70	3.42 (5.0)	5.31	5.35			
**6**	6.47 (6.0) ($J_{1,3}$ 1.2)	4.85 (3.2)	5.34 (5.6)	5.23 (7.4)	4.26 (5.4)	4.41 (11.8)	4.20 (3.1)
**7***	5.95 (5.5)	4.07	5.29	5.06 (9.7)	4.52	4.30 (12.5)	3.97 (2.2)
**8***	5.81 (1.4)	4.37 (3.7)	5.32 (9.7)	5.38 (9.7)	4.42	4.26	
**9***	5.67 (2.1)	3.83 (4.4)		5.40	4.27		
**16a**	6.44 (3.5)	3.58 (12.0)	5.37 (3.1)	5.40	4.28 (6.8)	4.10–4.00	4.10–4.00
**16b**	5.70 (9.5)	3.40 (12.0)	4.79 (3.2)	5.28	3.85 (6.6)	4.13–4.01	4.13–4.01
**16c**	6.47	3.48 (5.2)	5.38	5.41	4.35 (6.8)	4.13–4.01	4.13–4.01

* NMR data for compounds **3**, **4**, **5**, were obtained from the spectra of mixture containing compounds **2**–**5** (and **7**–**9**, respectively)

Table S6. $^{13}C\{^{1}H\}$-NMR data for compounds **1-9**, **16** ($CDCl_3$)

**Compound**	**C1**	**C2**	**C3**	**C4**	**C5**	**C6**
**1**	145.4	98.8	63.8	63.7	72.8	61.9
**2**	84.2	58.9	71.4	67.3	69.1	61.7
**3***	83.0	59.8	68.4	65.9	69.8	61.9
**4***	91.0	43.4	69.8	67.6	69.2	61
**5***	91.7	46.0	66.7	66.2		
**6**	145.8	99.1	67.6	67.3	74.2	61.6
**7***	83.7	62.3	72.9	68.4	70.2	61.8
**8***	82.5	63.3	71.5	65.9	71.3	62.1
**9***	90.2	47.1		66.7	74.0	
**16a**	92.9	43.0	69.3	67.2	68.5	61.4
**16b**	93.6	42.5	69.9	66.3	71.4	61.2
**16c**	95.8	45.1	65.8	66.5	69.0	61.4

* The signals of the compounds were obtained from the NMR spectra of mixtures containing them.

2.2 NMR Spectra of Crude Reaction Mixtures (Tables 1, S1, 2, 3)

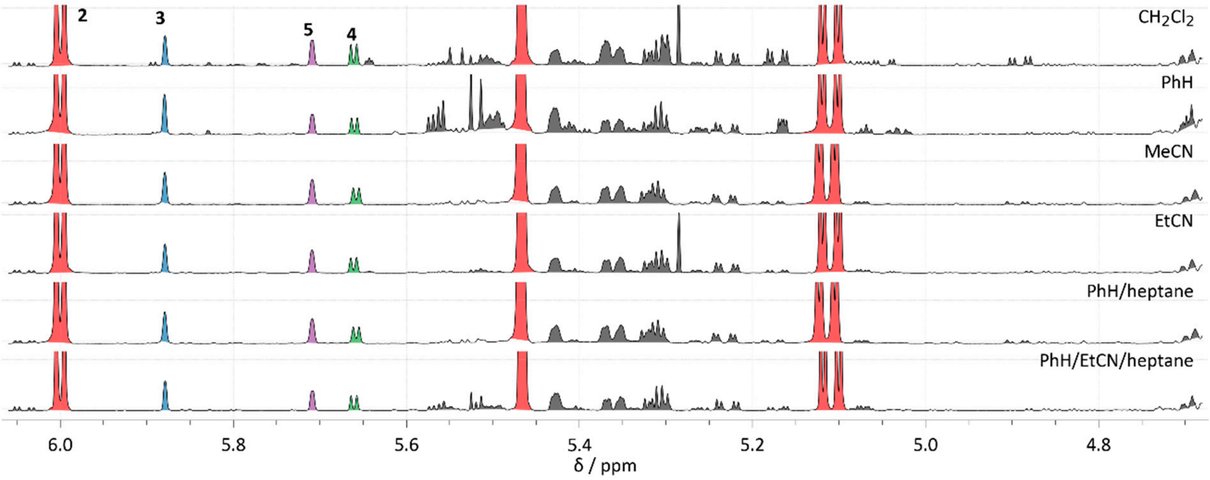

Figure S1. Comparison of ^{1}H NMR spectra of crude mixtures of APS transformation of galactal **1** in various solvents (Table 1) recorded in $CDCl_3$. Spectra are normalized to the area of the H-1 peak of the product **2** at δ 6.00 ppm. Labels **2-5** point out the peaks corresponding to H-1 of compounds **2-5** respectively. The areas under the peaks are color-coded: **2** (red), **3** (blue), **4** (green), **5** (purple), and unanalyzed impurities (gray).

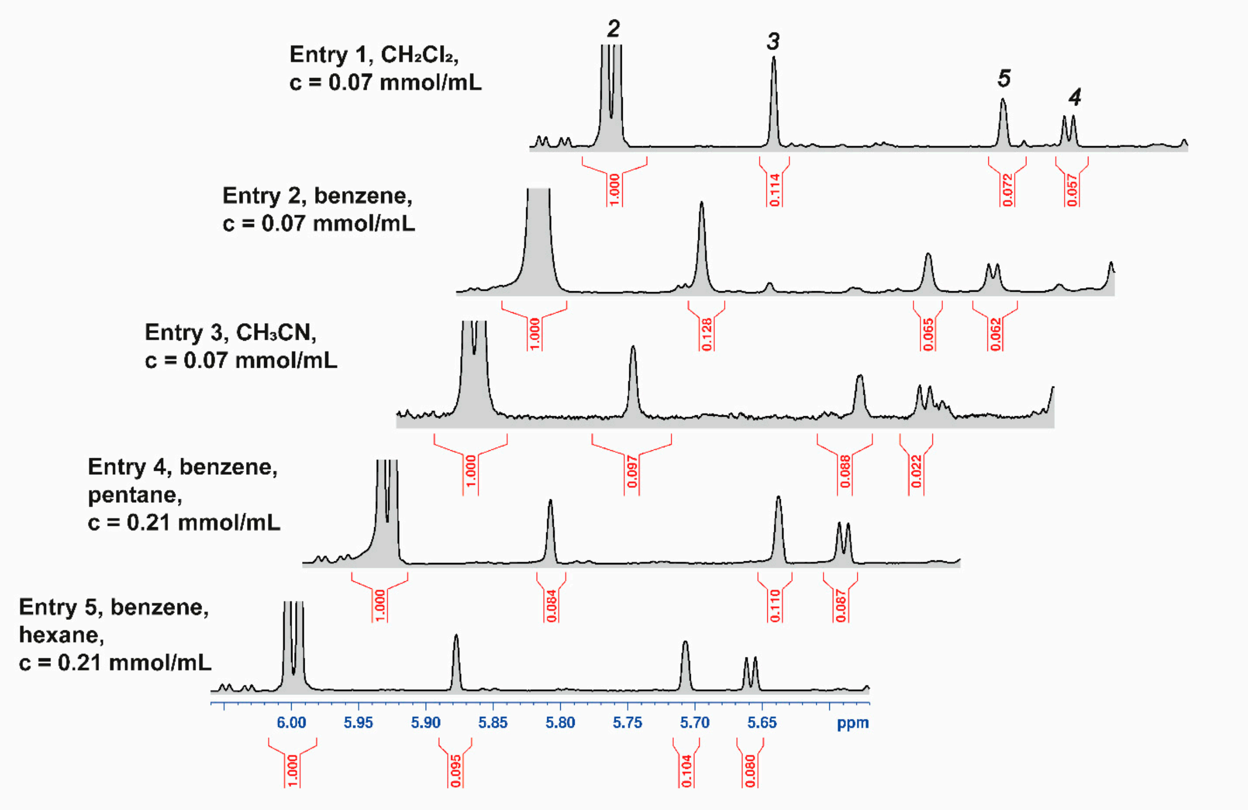

Figure S2. Selected ^{1}H NMR spectral region (δ 5.47–6.06 ppm) of the crude reaction mixtures of tri-acetylgalactal **1** APS (Table S1). The H-1 signals of compounds **2**, **3**, **5** and **4** are indicated by their respective labels.

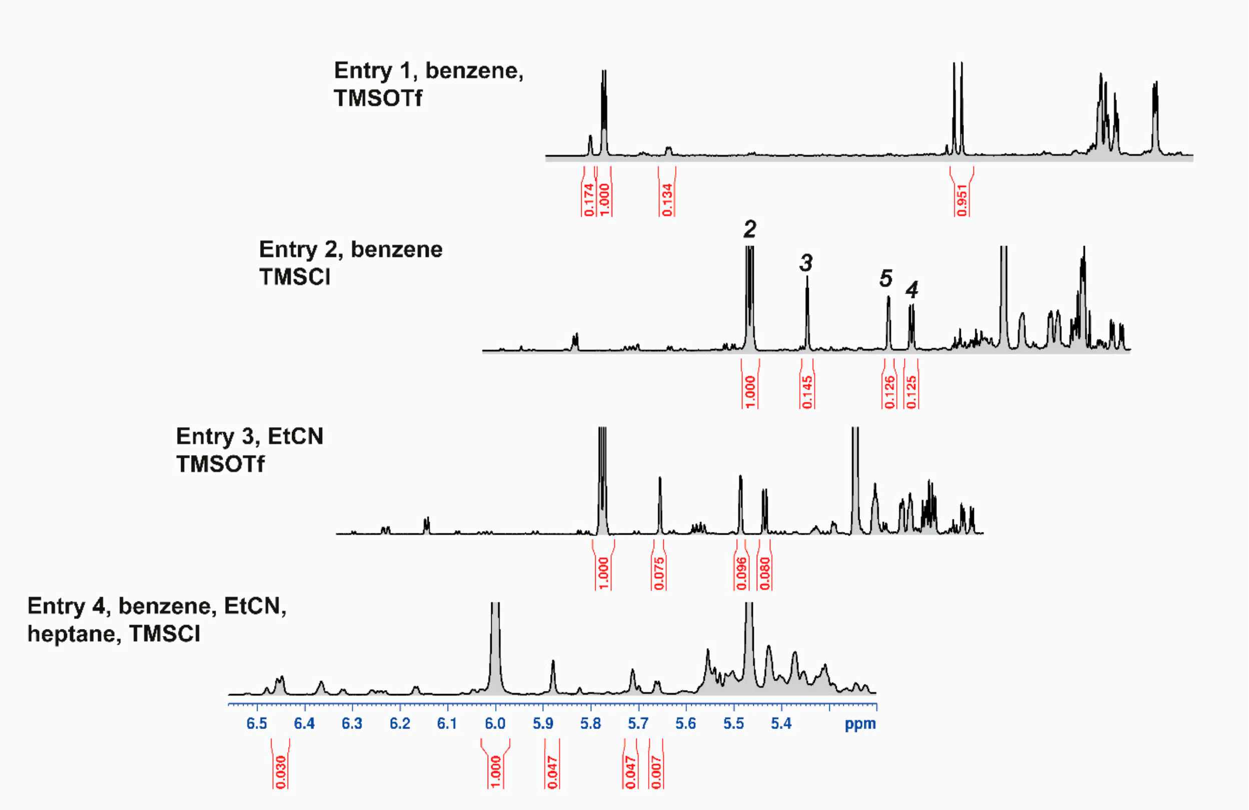

Figure S3. Selected ^{1}H NMR spectral region (δ 5.47–6.06 ppm) of the crude reaction mixtures of tri-acetylgalactal **1** APS (Table 2). The H-1 signals of compounds **2**, **3**, **5** and **4** are indicated by their respective labels.

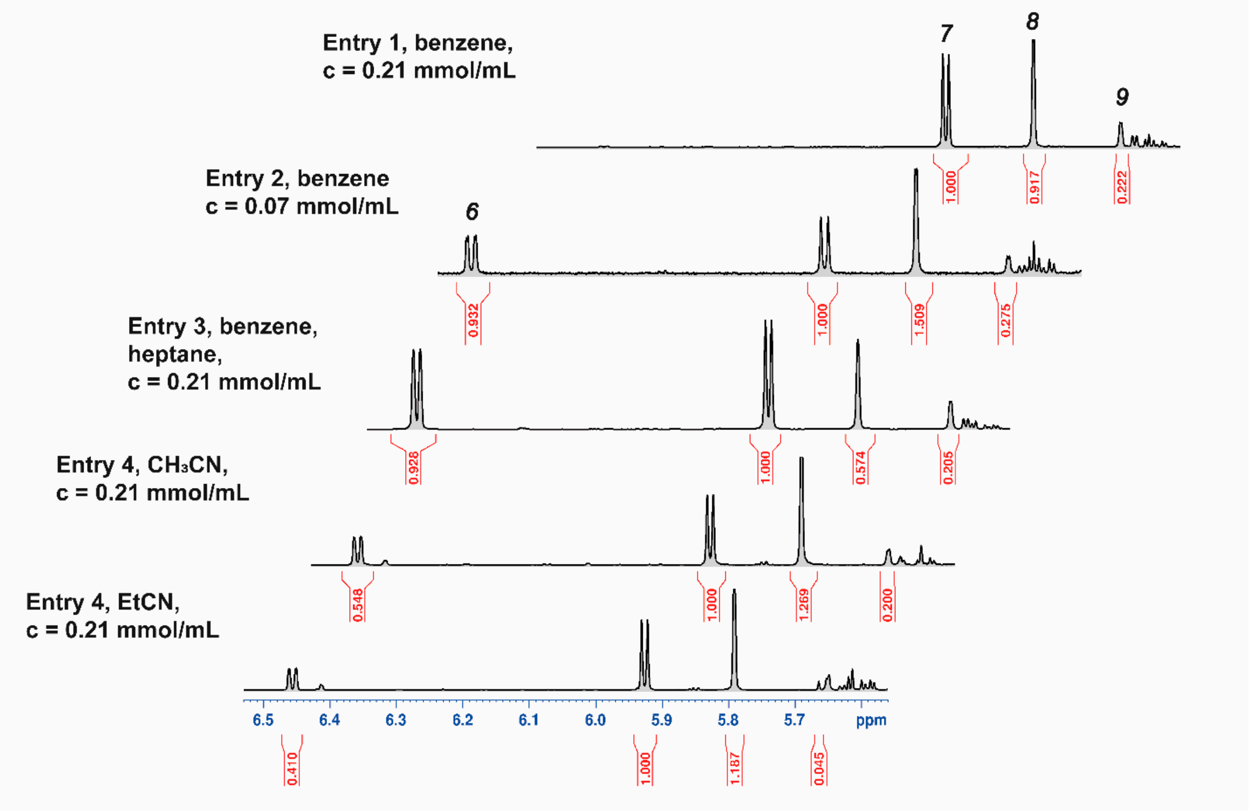

Figure S4. Selected ^{1}H NMR spectral region (δ 5.47–6.06 ppm) of the crude reaction mixtures tri-acetylglucal **6** APS (Table 3). The H-1 signals of compounds **6**, **7**, **8** and **9** are indicated by their respective labels.

3. References

1. Chang, C.; Wu, C.; Lin, M.; Liao, P.; Chang, C.; Chuang, H.; Lin, S.; Lam, S.; Verma, V.P.; Hsu, C.; et al. Establishment of Guidelines for the Control of Glycosylation Reactions and Intermediates by Quantitative Assessment of Reactivity. *Angew. Chem. Int. Ed.* **2019**, *58*, 16775–16779, doi:10.1002/anie.201906297.
2. Koseki, Y.; Watanabe, T.; Kamishima, T.; Kwon, E.; Kasai, H. Formation of Five-Membered Carbocycles from D -Glucose: A Concise Synthesis of 4-Hydroxy-2-(Hydroxymethyl)Cyclopentenone. *Bull. Chem. Soc. Jpn.* **2019**, *92*, 1324–1328, doi:10.1246/bcsj.20190063.
3. Grugel, H.; Albrecht, F.; Boysen, M.M.K. *Pseudo* Enantiomeric Carbohydrate Olefin Ligands – Case Study and Application in Kinetic Resolution in Rhodium(I)-Catalysed 1,4-Addition. *Adv. Synth. Catal.* **2014**, *356*, 3289–3294, doi:10.1002/adsc.201400459.
4. Dixon, J.T.; Van Heerden, F.R.; Holzapfel, C.W. Preparation of an Analogue of Orbicuside A, an Unusual Cardiac Glycoside. *Tetrahedron Asymmetry* **2005**, *16*, 393–401, doi:10.1016/j.tetasy.2004.11.028.
5. Nitz, M.; Bundle, D.R. Synthesis of Di- to Hexasaccharide 1,2-Linked β-Mannopyranan Oligomers, a Terminal S-Linked Tetrasaccharide Congener and the Corresponding BSA Glycoconjugates. *J. Org. Chem.* **2001**, *66*, 8411–8423, doi:10.1021/jo010570x.
6. Tokatly, A.I.; Vinnitsky, D.Z.; Kamneva, A.A.; Yashunsky, D.V.; Tsvetkov, Y.E.; Nifantiev, N.E. Glycosylation with Derivatives of Phenyl 2-Azido-2-Deoxy-1-Seleno-α-d-Gluco- and -α-d-Mannopyranosides. *Russ. Chem. Bull.* **2023**, *72*, 785–792, doi:10.1007/s11172-023-3842-6.
7. Guberman, M.; Pieber, B.; Seeberger, P.H. Safe and Scalable Continuous Flow Azidophenylselenylation of Galactal to Prepare Galactosamine Building Blocks. *Org. Process Res. Dev.* **2019**, *23*, 2764–2770, doi:10.1021/acs.oprd.9b00456.
